# Pragmatic Perspective on Conservation Genetics and Demographic History of the Last Surviving Population of Kashmir Red Deer (*Cervus elaphus hanglu*) in India

**DOI:** 10.1371/journal.pone.0117069

**Published:** 2015-02-11

**Authors:** Mukesh, Ved P. Kumar, Lalit K. Sharma, Malay Shukla, Sambandam Sathyakumar

**Affiliations:** 1 Wildlife Institute of India, Chandrabani, Dehradun 248 001, Uttarakhand, India; 2 Gujarat Forensic Sciences University, Gandhinagar 382007, Gujarat, India; Sichuan University, CHINA

## Abstract

The *hangul* (*Cervus elaphus hanglu*) is of great conservation concern because it represents the easternmost and only hope for an Asiatic survivor of the red deer species in the Indian subcontinent. Despite the rigorous conservation efforts of the Department of Wildlife Protection in Jammu & Kashmir, the *hangul* population has experienced a severe decline in numbers and range contraction in the past few decades. The *hangul* population once abundant in the past has largely become confined to the Dachigam landscape, with a recent population estimate of 218 individuals. We investigated the genetic variability and demographic history of the *hangul* population and found that it has shown a relatively low diversity estimates when compared to other red deer populations of the world. Neutrality tests, which are used to evaluate demographic effects, did not support population expansion, and the multimodal pattern of mismatch distribution indicated that the *hangul* population is under demographic equilibrium. Furthermore, the *hangul* population did not exhibit any signature of bottleneck footprints in the past, and Coalescent Bayesian Skyline plot analysis revealed that the population had not experienced any dramatic changes in the effective population size over the last several thousand years. We observed a strong evidence of sub-structuring in the population, wherein the majority of individuals were assigned to different clusters in Bayesian cluster analysis. Population viability analysis demonstrated insignificant changes in the mean population size, with a positive growth rate projected for the next hundred years. We discuss the phylogenetic status of *hangul* for the first time among the other red deer subspecies of the world and strongly recommend to upgrade *hangul* conservation status under IUCN that should be discrete from the other red deer subspecies of the world to draw more conservation attention from national and international bodies.

## Introduction

The *hangul*, or Kashmir red deer (*Cervus elaphus hanglu*), belongs to the Family Cervidae and is of immense conservation importance because it is the only subspecies of red deer in the Indian subcontinent that is endemic to the State of Jammu & Kashmir (J&K). The *hangul* population has experienced several challenges in the past due to environmental and anthropogenic pressures that have negatively influenced the population and resulted in severe decline in numbers and contraction in their distribution range [1, 2, 3]. Historically, the *hangul* was distributed in the mountains of Kashmir Himalaya, Chenab Valley and some parts of Chamba district in Himachal Pradesh [1]. However, there is only one viable population left today in the wild, which is largely confined to the Greater Dachigam Landscape (*ca*. 1,000 km^2^), encompassing the Dachigam National Park (NP) and adjoining Protected Areas (PA) [2, 3]. It is listed under Schedule-I of the Indian Wildlife (Protection) Act, 1972 and J&K Wildlife (Protection) Act, 1978 and has also been listed among the top 15 species of high conservation priority by the Government of India. However, the IUCN has not given any special status to this subspecies of red deer and has listed it as ‘Least Concern’, along with the other red deer subspecies of the world. The *hangul* population that once numbered 3,000 to 5,000 individuals around the year 1900 had been dramatically reduced to 700 individuals by 1987 [4]. A 1994 census found that the *hangul* population had been further reduced to 120 individuals. However, stringent conservation efforts with statistically robust population estimates showed a steady growth to 375 individuals by 2002, which then declined to 212 individuals in 2003 [4]. Thus, the *hangul* population has shown a fluctuating trend over the past few decades, with the most recent population size of 218 individuals (±13.96) estimated in 2011 [5]. Unfortunately, there has been a significant imbalance in the male-female and fawn-female ratios, which is vital for the long-term maintenance of the demographic structure of a healthy population. Recent estimates observed 26.52 males per 100 females and 27.52 fawns per 100 females [5]. Systematic research on the genetic makeup and demographic structure of the existing *hangul* population is crucial to suggest an effective management plan and recovery strategies. Studies have indicated that genetically isolated or small populations are at greater risk of extinction, not only due to threats posed by anthropogenic factors but also due to the vulnerability to loss of genetic diversity, inbreeding and stochastic processes [6, 7, 8]. To devise adequate conservation and management strategies, it is important to incorporate a reliable understanding of their genetic diversity and demographic history [9, 10], and molecular markers are important tools for analyzing demographic responses of populations and other historical processes [11, 12]. Nuclear microsatellite loci and mitochondrial DNA sequence data are complementary to each other in revealing multiple aspects of genetic diversity, demographic history and predicting genetic fitness for the survival and monitoring the wild populations [13, 14].

The previous events (i.e., severe reduction in numbers and shrinkage in the distribution range) raised concerns for the future survival of the *hangul* population in Dachigam. In this study, we investigated the population demography to understand whether the population reduction led to a severe bottleneck before the population expansion under the stringent protection measures of the Department of Wildlife Protection in J&K. We also examined how the historical events have shaped the current observable pattern of *hangul* population. In addition, we conducted a population viability analysis to predict the fate of survival of the *hangul* population in the Dachigam landscape for the next hundred years.

## Materials and Methods

### Ethical Statement

Ethical approval was not required for this study because all samples were noninvasively collected. Samples consisted of pneumatic hairs, which *hangul* as like other red deer species often shed during the onset of spring that act as an insulator by protecting them from severe cold during winters.

### Sample collection and DNA extraction

Shed hair samples of the *hangul* population were collected opportunistically from different parts of the Dachigam landscape during the systematic field study (2007 to 2012) conducted on the ecology of Asiatic black bear (*Ursus thibetanus*) by the Wildlife Institute of India and the three *hangul* population estimation exercises (2008, 2009 and 2011). In total, we collected 160 individual tufts of pneumatic hairs from different locations of the Dachigam NP. Details of sample collection and DNA extraction from hair samples have been presented elsewhere [15].

### PCR amplification and sequencing of D-loop region

Deer-specific primers were used to amplify the hyper-variable D-loop region of mitochondrial DNA of approximately 454 bp [16]. PCRs were conducted in 10 μl reaction mixtures that consisted of 0.25 mM MgCl_2_, 1 μM each of forward and reverse primers, 0.25 mM dNTPs and 1 unit *Taq* polymerase. Cycling conditions of PCR consisted of initial denaturation at 94°C for 2 min, 35 cycles of denaturation at 94°C for 30 seconds, an annealing step at 52°C for 20 seconds, an extension carried out at 72°C for 60 seconds and a final extension at 72°C for 10 min. The products were processed for cycle sequencing PCR using the Big Dye Terminator Cycle Sequencing Kit version 3.1 (Applied Biosystems, Foster City, CA, USA), and bi-directional sequencing was performed on an ABI 3130 Genetic Analyzer (Applied Biosystems, Foster City, CA,USA).

### PCR amplification and nuclear microsatellite genotyping

Six polymorphic microsatellite loci (Ca-13, Ca-18, Ca-30, Ca-38, Ca-42 and Ca-43) were amplified in this study, which were originally isolated from Chital deer (*Cervus axis*) and have been frequently used in several other cervids [17]. See [15] for details of multiplexing, thermal cycling profiles and individual identification from hair samples using these microsatellites.

## Data Analysis

### (a). Mitochondrial D-loop region

**Investigating genetic variability and demographic history**. All high-quality sequences were manually checked nucleotide by nucleotide using the Sequencher version 4.7 software (www.genecode.com). The cleaned sequences were subjected to multiple sequence alignments using CLUSTAL W, as implemented in the BioEdit version 7.2.5 software (http://www.mbio.ncsu.edu/BioEdit/bioedit.html), and trimmed to generate similar lengths of sequences for further analysis.

The number of haplotypes (H), nucleotide diversity (π), haplotype diversity (Hd), average number of nucleotide differences (K) and mismatch distribution test for demographic expansion, equilibrium or bottleneck [18] were computed using the program DnaSP version 5.10 [19]. Unimodal distributions are typical of populations that have experienced a recent expansion, whereas ragged and multimodal distributions are found in populations at demographic equilibrium [16].

Neutrality tests (i.e., Tajima’s D [20], Fu’s Fs [21] and Fu and Li’s F and D [22]) were also carried out to evaluate the demographic effects using DnaSP version 5.10 [19]. In addition, Harpending’s raggedness index (Rg) and sum of squared deviations (SSD) were calculated to test for demographic expansion under the sudden expansion model using Arlequin version 3.5.1.2 [23].

The history of the *hangul* population across time was reconstructed through the Coalescent Bayesian Skyline method [24], which provides estimates of changes in the effective population size over time/generations following the program BEAST version 2.1.3 [25]. We chose the HKY and empirical base frequencies under the substitution model, and the Markov Chain Monte Carlo (MCMC) repetitions were run for 10^8^ steps and yielded effective sample sizes (ESS) under a strict molecular clock and a stepwise skyline model. The molecular evolution was calibrated assuming a substitution rate of 0.118 × 10^–6^ substitutions/site/year, following [26, 27]. All operators were optimized automatically, and the results of the analysis were visualized using Tracer version 1.6 [28].

**Inference of geospatial genetic structure**. To evaluate the extent of population subdivisions, we used 13 sampling locations, considering a minimum of two or more individuals to be the unit of analysis (please see S1 Table). Analysis of molecular variance (AMOVA) was performed in Arlequin version 3.5.1.2 [23]. Furthermore, geospatial genetic structuring was examined by phylogenetic network employing the median-joining method [29], using the software Network version 4.5.1 (http://www.fluxusengineering.com) and assigning equal weights to all variable sites and with default values for the epsilon parameter (epsilon = 0).

**Molecular phylogenetic reconstruction**. The molecular phylogeography of red deer populations has been well studied across the world [27, 30, 31, 32, 33]. However, the non-availability of *hangul* sequences in the previous studies has left a large gap in understanding the phylogenetic status and delineating taxonomic boundaries of the *hangul* population among the other red deer subspecies of the world. We downloaded sequences of other red deer subspecies (please see S2 Table) that were complementary to the sequences generated in this study, and the phylogenetic relationship among red deer subspecies was reconstructed considering musk deer *(Moschus moschiferus*) as an outgroup. The maximum likelihood method and Tamura 3 parameter, the most fit substitution model for these sequences, were considered to reconstruct the phylogenetic tree using the Mega version 5.0 program [34].

### (b). Microsatellites

Allelic data were generated for six polymorphic microsatellites [15] and subjected to further analysis.

**Estimation of effective population size**. The effective population size (N_e_) estimates were computed using linkage disequilibrium information based on point-in-time samples as implemented in program LDNE [35]. This method assumes that markers are neutral, unlinked, and population was closed. This method performs well in non-ideal populations with skewed sex ratio or nonrandom variance in reproductive success [36]. Alleles with very low frequencies can bias the analytical results; therefore, the analysis was performed after removing alleles with frequencies (P_crit_) lower than 0.05. Both the parametric and jackknife procedures were applied to construct 95% confidence intervals.

**Detection of bottleneck footprints**. To investigate the presence of any bottleneck footprints, we performed transient heterozygosity excess and mode‐shift tests using 1,000 simulations in the Bottleneck program version 1.2.02 [37]. Principally, in a population that has recently experienced a genetic bottleneck, the mutation drift equilibrium transiently disrupts, and the population exhibits relatively high heterozygosity at the majority of loci to HWE equilibrium heterozygosity (Heq) [8, 32, 38]. Thus, the transient heterozygosity excess test was undertaken following the two phase model (TPM) of the Wilcoxon sign‐rank test, which is known to be the best fit for studying variation at microsatellites with di-nucleotide repeats (all six microsatellites used in this study were di-nucleotide repeats). The second method, the mode‐shift indicator test of allele distribution, followed the principle that a population that has passed through a recent genetic bottleneck tends to lose rare alleles more often and consequently inflates the frequencies of common alleles [39].

**Inference of population genetic structure**. To complement the detection of geospatial substructure using mitochondrial D-loop sequences, we tested the population genetic structure using the Bayesian method as implemented in Structure version 2.3.3 [40]. We followed the admixture model and a model of correlated allele frequencies with a burn‐in period of 50,000 and 5,00,000 MCMC repetitions. Twenty independent replicates were run, considering that there were K populations (*K* = 1 to 8) without prior knowledge of sampling locations (NOPRIOR). The most likely value of *K* was determined logically by comparing the log likelihood estimates at different values of *K* and by the rate of change in the log probability of the data between successive *K* values (Delta *K* [41]) using the web server of Structure Harvester [42]. The clustering results of Structure were visualized over ClumpaK (http://clumpak.tau.ac.il/index.html), a web server that provides a full pipeline for clustering, summarizing and visualizing Structure results. Following the previous studies [8, 43], each individual was assigned to the inferred clusters using a threshold proportion of membership (q) (i.e., q ≥0.80), or the individual was determined to be admixed if q < 0.80.

### (c). Population viability analysis

To investigate the impact of complex interactions of demographic, environmental and genetic factors on the population viability, the predictions were made in VOTEX version 10 following 100 simulations [44, 45, 46]. We built two scenarios using the parameters available to *hangul* population with an initial population size of 218 ±13.96 [5], carrying capacity (K) of 300, considering the habitat availability, and mean inbreeding coefficient (F_IS_) 0.38 ± 0.15 for scenario-I [15] and 6.23 for scenario-II (Vortex default values). Due to the lack of data on the species’ general biological traits, we considered published data of other red deer populations [47]. In addition, information based on oral history from experienced wildlife staff working in Dachigam for the last 20–25 years was also considered. (Please see S4 Table for the parameters used to model the scenarios).

## Results

Of the 160 total hair samples, 96 samples did not yield DNA. This was either due to a lack of root in the shed hairs or because the processed hairs were too few in number to provide detectable DNA on an agarose gel. Among the remnant samples, 54 hair samples yielded PCR amplification, of which 42 samples (77%) produced high-quality sequences that were used for the analyses.

### Genetic variability, demographic history and geospatial genetic structure

**Fig 1:**
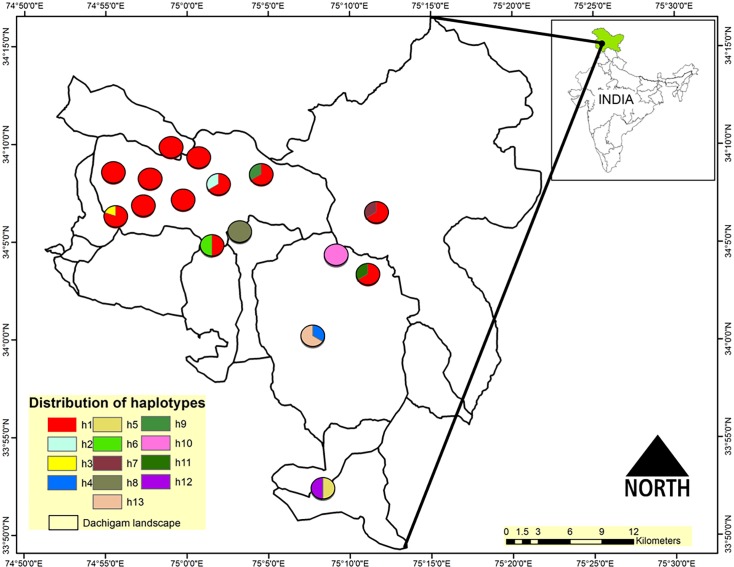
Map showing the positions of 16 sampling locations in the Dachigam landscape, Jammu & Kashmir, India.

In total, 42 sequences had 14 polymorphic sites, which resulted in 13 unique haplotypes with an average of 3.2 nucleotide differences. Of these, 27 individuals (64%) shared the same haplotypes (Hap 1), whereas 9 haplotypes (Hap 3 to Hap 9, Hap 11 and Hap 13) were found in one sample each (Table 1 and Fig. 1). All 13 novel haplotypes were deposited in the GenBank database (Accession no. KJ937024 to KJ937036). The overall haplotype (Hd) and nucleotide diversity (π) were 0.589 ± 0.091 and 0.008 ± 0.001, respectively (Table 2). The studied population showed a multimodal pattern of mismatch distribution, indicating the *hangul* population to be under demographic equilibrium (please see S1 Fig.). The estimates of neutrality tests in general showed the pattern revealed by the mismatch distribution curve (Table 2). The negative estimates of Tajima’s D and Fu’s Fs statistic tests showed that rare alleles are more common than expected. However, the observed estimates were not significant (*P* >0.5), suggesting that the population has not undergone an expansion. Fu and Li’s D and F tests also indicated no significant departure from neutrality (*P*>0.10). To assess the fit of our data, the mismatch distribution under demographic expansion was simulated with SSD and Rg, where a non-significant test indicates a good fit and support of expansion. Non-significant SSD values of the mismatch distribution supported a sudden expansion model. The Rg [48], based on mismatch distributions, was used to test whether the sequence data deviated from what is expected under a sudden expansion model, and we observed a marginal to non-significant raggedness index (Table 2).

**Table 1:** The nucleotide sequence differences and NCBI accession numbers of 13 haplotypes of mitochondrial D-loop region of *hangul* population.

**Haplotypes**	**Accession No.**	**Variable sites**													
		104	109	139	223	264	291	337	354	358	367	372	373	383	390
Hap- 1	KJ937024	C	T	T	A	A	G	A	C	G	T	C	T	T	T
Hap- 2	KJ937025	.	.	.	.	T	A	.	.	.	.	.	.	.	G
Hap- 3	KJ937026	.	.	.	.	.	.	.	.	.	.	.	.	.	G
Hap- 4	KJ937027	G	A	.	G	.	.	.	.	.	.	.	C	A	G
Hap- 5	KJ937028	G	A	.	G	.	.	C	.	A	.	.	C	A	G
Hap- 6	KJ937029	G	.	.	.	.	.	C	.	A	.	.	.	A	G
Hap- 7	KJ937030	G	A	G	.	.	.	C	.	A	.	.	C	A	G
Hap- 8	KJ937031	G	A	G	.	.	.	.	G	.	C	G	.	A	G
Hap- 9	KJ937032	G	.	.	.	.	.	.	.	.	C	G	.	A	G
Hap- 10	KJ937033	G	.	.	G	.	.	C	.	.	.	.	.	.	.
Hap- 11	KJ937034	G	.	.	G	.	.	.	.	.	.	.	.	.	.
Hap- 12	KJ937035	G	A	.	G	.	.	C	.	.	.	G	C	A	G
Hap- 13	KJ937036	G	A	G	G	.	.	C	.	A	.	.	C	A	G

**Table 2:** Summary of molecular genetic diversity and neutrality tests of demographic expansion of *hangul* population.

**Diversity estimates (D-loop)**						**Neutrality tests**				**Mismatch distribution**		**Diversity estimates†(microsatellites)**			**GDE**	**Effective population size at frequency (0.05)**		
N	P	H	K	Hd	π	Tajima’s D*	Fu’s Fs*	Fu and Li’s D*	Fu and Li’s F*	SSD*	Rg*	Ho	He	F_IS_	Heq	N_e_	95% CI (parametric)	95% CI (Jackknife)
42	14	13	3.20	0.589 ±0.091	0.008 ±0.001	-0.049	-2.598	1.075	0.831	0.076	0.191	0.40 ±0.11	0.66 ±0.66	0.38 ±0.15	0.763 ±0.033	20.5	9.4–86.1	8.8–115.8

N- Number of samples; P- Polymorphic sites; H- Number of Haplotypes; K- Average number of nucleotide differences; Hd-Haplotypes diversity; π- Nucleotide diversity; SSD- Sums of squared deviations; Rg- Harpending’s raggedness index; * *P* >0.10; Ho– observed heterozygosity; He- expected heterozygosity; F_IS_- inbreeding coefficient index; GDE- Gene diversity excess test under TPM of Wilcoxon signed‐rank test; † data from Mukesh *et al*. (2013) [15].

**Fig 2:**
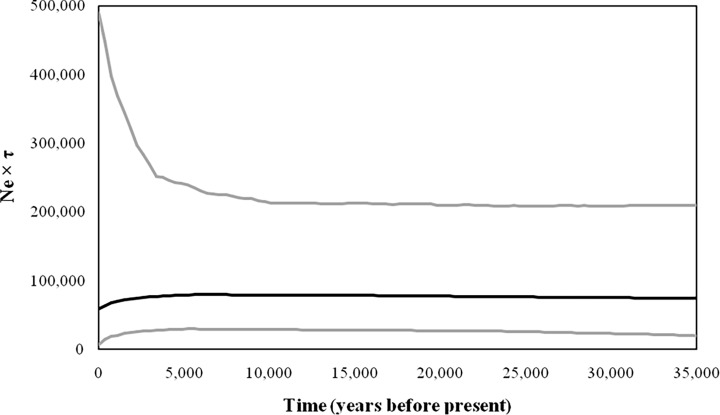
Demographic history of *hangul* population estimated using Bayesian skyline plot. Bayesian skyline plot showing an overall stable population size during recent past. The solid line shows the median estimates of Ne τ (Ne = effective population size; τ = generation time), and the grey lines around median estimates show the 95% highest posterior density (HPD) estimate of the historic effective population size. The timing of events was estimated assuming a substitution rate of 0.118 × 10^–6^ substitutions/site/year (Stoneking *et al*., 1992; Mahmut *et al*., 2002). The time is shown from 0 (present) to 35 (kya).

The Bayesian skyline plot revealed a slight but steady growth of the effective population size over the last 35,000 years (Fig. 2). Thus, the demographic scenario recovered in the BSP and the findings of neutrality tests and multimodal mismatch distribution curve corroborated each other, suggesting that there has been no evidence of dramatic changes in the effective population size over the last several thousand years.

**Fig 3:**
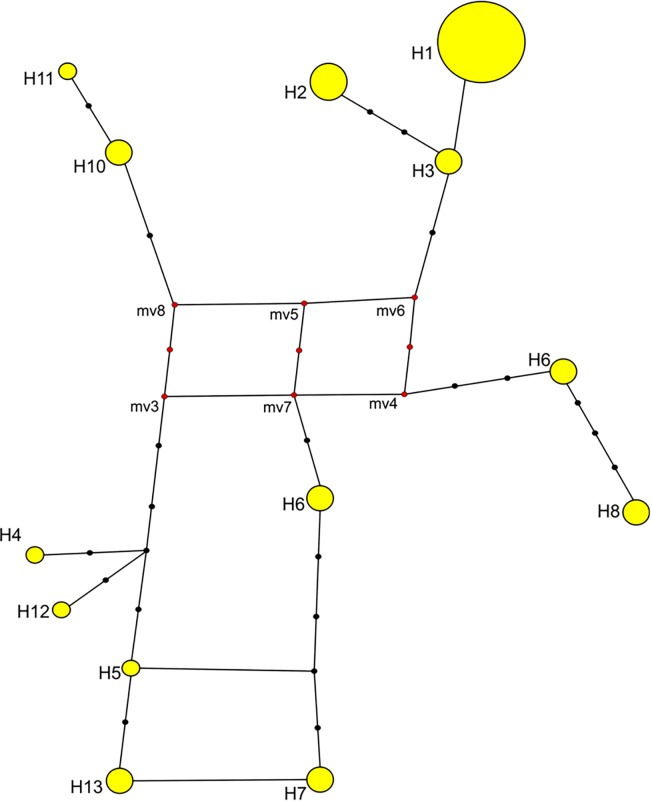
Median-joining network of *hangul* population. Size of circles represents the relative frequency of that haplotype present in the population.

The AMOVA showed that 87.31% of the molecular variance was found among sampling locations, whereas 12.69% was found within sampling locations (Table 3). Accordingly, the fixation index was high and significant (F_ST_ = 0.87, P< 0.001). The median-joining network analysis of 13 haplotypes suggested little geospatial structure and did not support a population expansion, as expansions are generally characterized by a star-like network radiating from a core and abundant haplotypes (Fig. 3).

**Table 3:** Analysis of molecular variance and Bayesian clustering analysis of *hangul* population.

**Source of variation**	**Degree of freedom**	**Sum of squares**	**Variance components**	**Percentage of variation**
Among location	4	51.710	2.59034 Va	87.31
Within locations	37	13.933	0.37658 Vb	12.69
Total	41	65.643	2.96691	F_ST_ = 0.87, *P* < 0.001
Bayesian clustering analysis of *hangul* population				
*Hangul* pop.	Cluster 1	Cluster 2	Assigned Individuals	Unassigned Individuals
30	0.47 (14)	0.33 (10)	0.80 (24)	0.20 (6)

**Fig 4:**
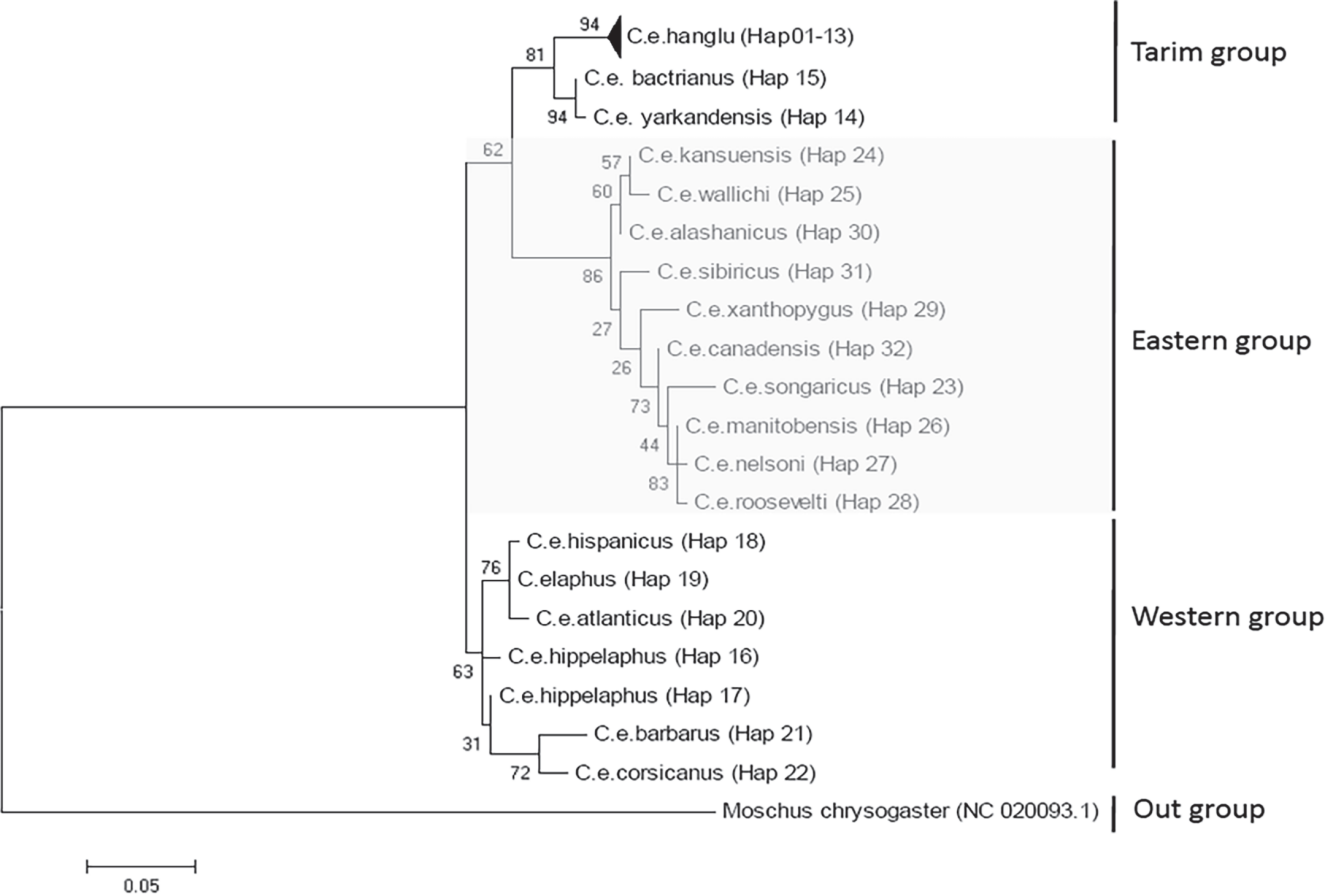
Reconstructing the phylogenetic relationships among subspecies of red deer with inclusion of *hangul* sequences of D-loop region following maximum likelihood method and Tamura 3 parameter distance matrix. Haplotype names refer to those given in S2 Table. Numbers near internal branches are bootstrap values (%) derived from 1,000 replications.

In the phylogenetic analysis, three distinct clusters were formed: cluster 1- Central Asian (Tarim group- *Hangul*, *Bactrian* and *Yarkand* red deer); cluster 2- North American red deer (Eastern group); and cluster 3- European red deer (Western group). The clusters showed 81, 86 and 63% bootstrap support, respectively and the results were in accordance with the previous study [27] (Fig. 4). All 13 haplotypes of the *hangul* population formed a subcluster within the Tarim group, with 94% bootstrap support, and were found to be genetically closer to Bactrian deer than Yarkand deer, which are also in the geographical proximity. We observed a 119-bp deletion in the Tarim group that was also reported in the Western group and indicated ancestral movements of individuals between the Tarim and Western populations. However, the Tarim group is geographically closer to the Eastern population (please see S3 Table).

### Effective population size, bottleneck footprints and population genetic structure

The *hangul* population displayed a stable effective population size (i.e., N_e_-20.5; 95% CI-9.4–86.1 [parametric] and 8.8–115.8 [Jackknife]), suggesting that the population still contains a sufficient number of breeders despite exhibiting low genetic diversity and inbreeding (Table 2).

The bottleneck investigation through a gene diversity excess test showed that the expected heterozygosity under equilibrium was higher than the heterozygosity observed (Table 2). Thus, the null hypothesis of mutation drift equilibrium was rejected, suggesting no apparent signature of population bottleneck in the recent past. The mode shift test also resulted in a normal “L”-shaped allele distribution curve, indicating the presence of a larger proportion of alleles at low allele frequency classes (please see S2 Fig.) and thus the lack of a genetic bottleneck in the *hangul* population.

**Fig 5:**
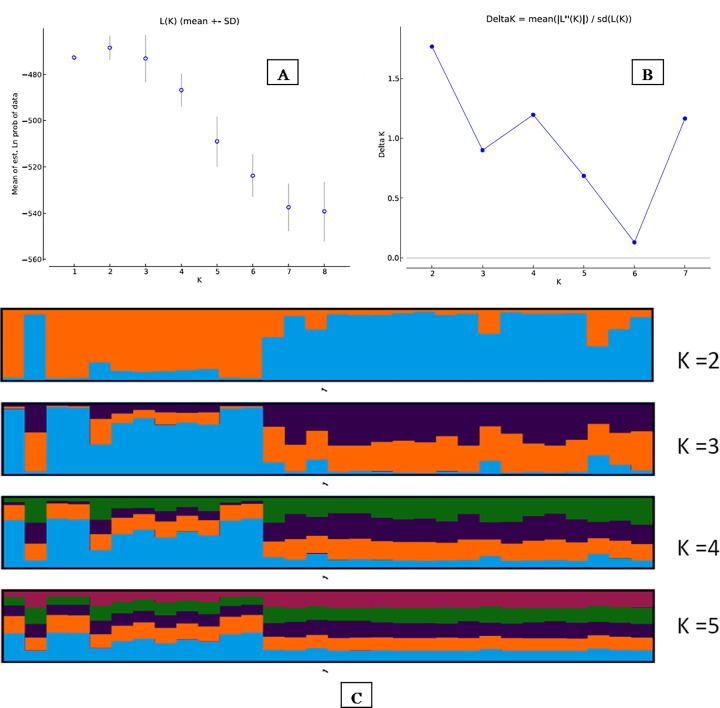
Bayesian clustering patterns of *hangul* population; (A) Mean L(K) (±SD) over 20 runs for each *K*-value; (B) ad hoc quantity (ΔK); (C) bar plots of individual assignments (K = 2 to 5).

We observed a multimodal curve in population assignment through the ad hoc quantity value, and the maximum value for the estimated mean likelihood of *K* (mean Ln P [X/*K* = -468.4850) was found at *K* = 2 (Fig. 5 A & B), considering the fact that the method implemented in Structure only permits an ad hoc approximation and the biological interpretation of *K* might not be straightforward [35]. Therefore, the best strategy is to always select the lowest value of *K* that captures the maximum degree of structure detected in the data. Furthermore, if some of the individuals are strongly assigned to one population, or if the proportions assigned to each group are asymmetrical, there is strong evidence that real population structure exists. We present structure outputs (*K* value at 2 to 5; Fig. 3C) but adopted *K* = 2 where the majority of the individuals (80%) were assigned (considering *q* value ≥0.80) in cluster1 (47%) and cluster2 (33%)), and only 20% of the individuals were unassigned at *q* value < 0.80.

### Population viability analysis

**Fig 6:**
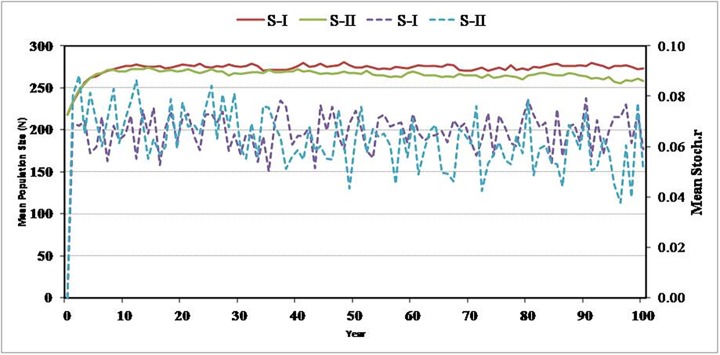
Population viability analysis of *hangul* population in the Dachigam landscape.

The simulations resulted in a mean population of 273 ± 2.2 individuals in scenario-I and 262 ± 2.5 individuals in scenario-II without the probability of extinction in the next hundred years. The mean growth rate (r) stochastic was positive and found to be 0.0665 ± 0.0007 and 0.0638 ± 0.0010 in scenario-I and scenario-II, respectively (Fig. 6). Both scenarios, with different levels of inbreeding depressions or total lethal equivalents per individuals, has demonstrated insignificant impacts in terms of change in population growth rate, mean population size and viability of the population.

Solid lines represent population size and dotted lines represent r stochastic growth rate in next 100 years in Scenario-I (S-I) and Scenario-II (S-II).

## Discussion

The present study is an extended validation of our previous study [15] in providing the facts that existing the *hangul* population in the Dachigam landscape showed a low genetic variation compared with the several red deer populations of the world: Ireland red deer (Hd 0.492–0.808 and π 0.00157–0.01639; [49]), Scottish red deer (Hd 0.2529–0.8364 and π 0.0006–0.0057; [50]), Carpathian red deer (Hd 0.61–0.81 and π 0.10–0.954; [51]) and Norwegian red deer (Hd 0.213–0.643 and π 0.004–0.0053; [52]). Please see [53] for a comprehensive review of the population genetics of the European red deer. Low genetic variability may indicate that the population is susceptible to the ill effects of inbreeding, which could affect fitness and local adaptability (due to the loss of rare alleles) with the change in the environment. However, detection of low genetic diversity in some species is a reported phenomenon, as shown in Beringian steppe bison [54], koalas, Tasmanian devils and Iberian lynxes [55], and *hangul* might provide a similar case as observed in the present study.

All tests of demographic analyses complemented each other. For mtDNA, the multimodal mismatch distribution curve indicates that the population is under demographic equilibrium and reflects that population did not undergo a bottleneck in the past. Neutrality tests applied to search for additional demographic signs of population expansion are also in concordance with the above pattern (Table 2). Additionally, no bottleneck event was observed at microsatellites, as indicated by the presence of an L-shaped allele distribution curve and the occurrence of higher-than-expected heterozygosity under equilibrium than the heterozygosity observed under the gene diversity excess test (Table 2). The Bayesian skyline plot analysis provided apparent evidence of a slight reduction in effective population size and strongly indicated that the population has been demographically stable over the last 35,000 years (Fig. 2). This study highlights the unreliability of *hangul* population estimates made during 1900 (~ 3000–5000 individuals), which was followed by a drastic decline to 120 individuals in a 1994 census and a most recent estimate of 218 individuals in 2011. If the population estimates of 1900 were accurate, then the population must have experienced a severe genetic bottleneck in the past. However, this was not observed in the present study, and the *hangul* population, conversely, was found to be stable in the past several thousand years. This result indicated that prior population estimates (before 2004) may not be reliable as they were not conducted using scientific census methods and proper sampling design. Regardless of the low diversity estimates, the *hangul* population appears to contain a sufficient number of effective breeders. However, N_e_ estimates should be interpreted with caution, as the different methods may give considerably different results and can be biased with the number of samples and marker used [30, 56]. The results demonstrated that the existing population has inherent potential/vigor to recover from the current scenario given stringent habitat protection and other conservation measures.

The AMOVA and median-joining network analysis indicated a little geospatial structure, as there was high molecular variance among sampling locations in comparison to within sampling locations (Fig. 3). Bayesian cluster analysis revealed a substructure in the population, as the majority of the individuals (*ca*. 80%) were strongly assigned to one of two clusters, and only 20% of the individuals remained unassigned. This result strongly indicated the presence of a geospatial population structure, as the analyzed samples were representative of the entire reported distribution range of the *hangul* population in the Dachigam landscape.

The population viability analysis predicted that the current level of inbreeding will not have serious consequences on the long-term viability of the *hangul* population, and a positive growth rate indicated no sign of an expiration risk in the next hundred years. The positive trends in stochasticity growth rate (r) and the genetic history suggest that the *hangul* population will be able to cope with the current demographic situations if effective conservation efforts are made. Studies conducted on other large mammals indicate that populations with similar demographic histories have done quite well if genuine efforts are made towards protection of the species and enhancement of its habitat [57, 58]. This study is the first attempt to resolve the phylogenetic status and delineate the taxonomic boundaries of *hangul* among the other red deer subspecies, as no sequence of *C*. *e*. *hanglu* was available in NCBI/GenBank database prior to our submission. In phylogenetic analysis, all 13 haplotypes of the *hangul* population formed a subcluster within the Tarim group with 94% bootstrap support and were found to be genetically closer to Bactrian deer than Yarkand deer (Fig. 4). Interestingly, a 119bp deletion was observed in the Tarim and Western groups, indicating these two populations shared ancestral lineages in the past. However, the Tarim group was geographically closer to the Eastern population.

With context from available studies on *hangul*, it has been noted that the male-female and fawn-female ratios have drastically declined in the past. The most significant decline in fawn-female ratio was observed in 2006, with 9 fawns to 100 females [5]. This low recruitment rate could be attributed to poor protection leading to a severe decline in potential number of breeders or breeding failures when the individuals were under stress and/or possible misclassification errors during field observations. The intrusion of migratory graziers in the high-altitude areas of Dachigam NP during summer is also of great concern because they may lead to habitat encroachment, range contraction and poor survival rates due to grazing competition and disease transmittance from livestock. External forces, such as the ease of resource availability in lower Dachigam, salt lick sites, supplementing feed exercises by the Department of Wildlife Protection of J & K, lack of proper connectivity between relic populations (forms transiently during winter migration towards lower Dachigam) and the major population in Dachigam, collectively drive the individuals and cause colonization into small groups. This further increases the possibility of inbreeding between closely related individuals in the *hangul* population. Low genetic diversity and a decline in the effective number of breeders may lead to further population contraction and adverse effects of inbreeding, which could imperil the long-term survival of the *hangul* population at Dachigam NP.

## Management Recommendations

This study revealed that the *hangul* population in Dachigam has been stable through many thousands of years, and viability predictions for the next 100 years have shown that the population will remain stable with positive growth. Therefore, we presume that the *hangul* population has survived in this landscape since time immemorial without any genetic drift. The reduction of the population in the past was largely due to poor protection and insufficient management of its habitat. The current model of development is leading to the fragmentation of forested habitat in the Dachigam landscape, leading in turn to the deterioration of the corridor functionality, which adversely hampers the animal movement that is pivotal to maintaining genetic diversity and gene flow [59]. Populations that are completely isolated or exist in small numbers are more vulnerable to genetic erosion than the contiguous populations or populations that exist in large numbers [58]. For instance, small groups of *hangul* with few individuals that were reported in the southernmost corner of Dachigam in *Sikhargah* [2] are most likely most vulnerable to extirpation because of anticipated fragmentation and habitat loss, leading to no/marginal gene flow. We foresee that conservation efforts should emphasize mapping, protecting and enriching those important forest patches where potential *hangul* habitat still remains in the landscape. The endangered Asiatic lions (*Panthera leo persica*) in Gir Forests, Gujarat, India was recovered after a severe reduction in population size (177 individuals left in wild during 1968) because of effective protection and informed management actions [60]. Similar approaches can be followed for the *hangul* recovery, as the present study has shown that the *hangul* population retains its genetic potential and can bounce back to its historic status if wise efforts are made with respect to proper protection enforcement and habitat quality enhancement.

The IUCN World Conservation Union has recognized the importance of the captive propagation programs for any taxon whose wild population declines below one thousand individuals [61]. Therefore, we suggest that conservation breeding should be initiated as insurance against extinction and to recruit new individuals into the population. However, it is also pertinent to mention here that the augmentation of *hangul* population (conservation breeding and reintroduction) could be taken up only after strengthening the *in-situ* conservation and management efforts and when the current threats to *hangul* are completely addressed. The present study has examined the realistic fate and explored the possibility of recovering the *hangul* population through stringent management efforts. The findings of the present study would be of immense utility to decision/policy makers and park managers who may prioritize the appropriate strategies for the implementation of the species recovery plan with more realistic facts and procedures.

In view of its conservation status, the *hangul* is listed in Appendix I of the Convention on International Trade in Endangered Species of Wild Fauna and Flora (CITES), which forbids the trade of this species and highlights ongoing conservation priorities and efforts. However, the IUCN has not considered giving any special status to this subspecies of red deer. Here, we strongly question why this subspecies is not listed under the IUCN but is instead merged with the red deer species complex as Least Concern. Whereas every subspecies of tiger (*Panthera tigris*) has been assessed separately, the red deer subspecies have been lumped into a single conservation category of Least Concern under the IUCN conservation status. Considering the low genetic variability, imbalanced sex ratio, low recruitment rate and reduction in distribution range and population size, we strongly recommend that the status of *hangul* should be upgraded from ‘Least Concern’ to ‘Endangered’ by the IUCN to seek the attention of national and international bodies to work towards the conservation and long-term survival of this subspecies of red deer.

## Supporting Information

S1 Table Geographical distribution of 13 haplotypes of mitochondrial D-loop region of *hangul* population. (DOCX)

S2 Table List of haplotypes, origin and their GenBank accession no. of red deer subspecies used in reconstructing phylogeny with inclusion of *hangul* haplotypes. (DOCX)

S3 Table Variable sites in mitochondrial D-loop region sequences with respect to complete mitochondrial genome of red deer (GenBank accession no. NC-007704.2). (DOCX)

S4 Table Summary of parameters used to model the scenarios; EV = environmental variation, expressed as a standard deviation. (DOCX)

S1 Fig Pairwise mismatch distribution using mitochondrial D-loop sequences of *hangul* population. (DOCX)

S2 Fig Qualitative L‐shaped mode‐shift allele distribution test for bottleneck analysis of *hangul* populations. (DOC)
