# Supplementary material for: Pragmatic Perspective on Conservation Genetics and Demographic History of the Last Surviving Population of Kashmir Red Deer (Cervus elaphus hanglu) in India
Source: PLoS One. 2015 Feb 11;10(2):e0117069. doi: 10.1371/journal.pone.0117069 (PMC4324630; doi:10.1371/journal.pone.0117069)
Supplement: S1 Table — (DOCX) [file pone.0117069.s001.docx]

**Supporting table S1 - Geographical distribution of 13 hapotypes of mitochondrial D loop region of *hangul* population**

| **Haplotypes** | **Sampling locations†** | | | | | | | | | | | | | | | | |
| --- | --- | --- | --- | --- | --- | --- | --- | --- | --- | --- | --- | --- | --- | --- | --- | --- | --- |
|  | **1** | **2** | **3** | **4** | **5** | **6** | **7** | **8** | **9** | **10** | **11** | **12** | **13** | **14** | **15** | **16** | **N** |
| Hap- 1 | 1 | 2 | 4 | 1 | 4 | 3 | 3 | 2 |  | 2 |  | 2 | 1 | 2 |  |  | 27 |
| Hap- 2 |  |  | 2 |  |  |  |  |  |  |  |  |  |  |  |  |  | 2 |
| Hap- 3 |  |  |  |  | 1 |  |  |  |  |  |  |  |  |  |  |  | 1 |
| Hap- 4 |  |  |  |  |  |  |  |  |  |  |  |  |  |  | 1 |  | 1 |
| Hap- 5 |  |  |  |  |  |  |  |  |  |  |  |  |  |  |  | 1 | 1 |
| Hap- 6 |  |  |  |  |  |  |  |  |  |  |  |  | 1 |  |  |  | 1 |
| Hap- 7 |  |  |  |  |  |  |  |  |  |  |  |  |  | 1 |  |  | 1 |
| Hap- 8 |  |  |  |  |  |  |  |  | 1 |  |  |  |  |  |  |  | 1 |
| Hap- 9 |  |  |  |  |  |  |  | 1 |  |  |  |  |  |  |  |  | 1 |
| sHap- 10 |  |  |  |  |  |  |  |  |  |  | 2 |  |  |  |  |  | 2 |
| Hap- 11 |  |  |  |  |  |  |  |  |  | 1 |  |  |  |  |  |  | 1 |
| Hap- 12 |  |  |  |  |  |  |  |  |  |  |  |  |  |  |  | 1 | 1 |
| Hap- 13 |  |  |  |  |  |  |  |  |  |  |  |  |  |  | 2 |  | 2 |
| **Total** | **1** | **2** | **6** | **1** | **5** | **3** | **3** | **3** | **1** | **3** | **2** | **2** | **2** | **3** | **3** | **2** | **42** |

**†** Sampling locations are shown in figure 1; thirteen sampling locations were used for analysis of molecular variance (AMOVA) and computing a general F_ST_ value considering two or more individuals as the units of analysis (locations with only one individual pooled with the nearest sampling location for analysis)
