# Supplementary material for: Pragmatic Perspective on Conservation Genetics and Demographic History of the Last Surviving Population of Kashmir Red Deer (Cervus elaphus hanglu) in India
Source: PLoS One. 2015 Feb 11;10(2):e0117069. doi: 10.1371/journal.pone.0117069 (PMC4324630; doi:10.1371/journal.pone.0117069)
Supplement: S2 Table — (DOCX) [file pone.0117069.s002.docx]

**Supporting table S2 – List of haplotypes, origin and their GenBank accession no. of red deer subspecies used in reconstructing phylogeney with inclusion of hangul haplotypes**

| **Haplotypes** | **Subspecies** | **Origin** | **Group** | **GenBank accession nos.** |
| --- | --- | --- | --- | --- |
| Hap-01**†** | *C. e. hanglu* | J&K | Tarim | KJ937024 |
| Hap-02**†** | *C. e. hanglu* | J&K | Tarim | KJ937025 |
| Hap-03**†** | *C. e. hanglu* | J&K | Tarim | KJ937026 |
| Hap-04**†** | *C. e. hanglu* | J&K | Tarim | KJ937027 |
| Hap-05**†** | *C. e. hanglu* | J&K | Tarim | KJ937028 |
| Hap-06**†** | *C. e. hanglu* | J&K | Tarim | KJ937029 |
| Hap-07**†** | *C. e. hanglu* | J&K | Tarim | KJ937030 |
| Hap-08**†** | *C. e. hanglu* | J&K | Tarim | KJ937031 |
| Hap-09**†** | *C. e. hanglu* | J&K | Tarim | KJ937032 |
| Hap-10**†** | *C. e. hanglu* | J&K | Tarim | KJ937033 |
| Hap-11**†** | *C. e. hanglu* | J&K | Tarim | KJ937034 |
| Hap-12**†** | *C. e. hanglu* | J&K | Tarim | KJ937035 |
| Hap-13**†** | *C. e. hanglu* | J&K | Tarim | KJ937036 |
| Hap-14 | *C. e. yarkandensis* | Tarim | Tarim | AB074558 |
| Hap-15 | *C. e. bactrianus* | Tadzikistan | Tarim | AF296823 |
| Hap-16 | *C. e. hippelaphus* | Italy (South) | Western | AF291886 |
| Hap-17 | *C .e. hippelaphus* | Italy (North) | Western | AF291887 |
| Hap-18 | *C. e. hispanicus* | Spain, | Western | AF291889 |
| Hap-19 | *C. elaphus* | Unknown | Western | AF016972 |
| Hap-20 | *C. e. atlantics* | Norway | Western | AF291888 |
| Hap-21 | *C. e. barbarus* | Tunisia | Western | AF296808 |
| Hap-22 | *C. e. corsicanus* | Sardegna | Western | AF291885 |
| Hap-23 | *C. e. songaricus* | China | Eastern | KF781103 |
| Hap-24 | *C. e. kansuensis* | China | Eastern | GQ304767 |
| Hap-25 | *C. e. wallichi* | China, Tibet | Eastern | KF879776 |
| Hap-26 | *C. e.manitobensis* | China | Eastern | AF016957 |
| Hap-27 | *C. e. nelsoni* | China | Eastern | KF153099 |
| Hap-28 | *C. e. roosevelti* | Russia | Eastern | AF016970 |
| Hap-29 | *C. e. xanthopygus* | Mongolia | Eastern | GQ304774 |
| Hap-30 | *C. e. alashanicus* | China | Eastern | GQ304768 |
| Hap-31 | *C. e. sibiricus* | China | Eastern | GQ304769 |
| Hap-32 | *C. e. canadensis* | North America | Eastern | AY970666 |
| Out group | *Moschus chrysogaster* | ---- | ----- | NC 020093 |

**†** Sequences generated in this study and submitted to GenBank/NCBI.
