# Supplementary material for: Pragmatic Perspective on Conservation Genetics and Demographic History of the Last Surviving Population of Kashmir Red Deer (Cervus elaphus hanglu) in India
Source: PLoS One. 2015 Feb 11;10(2):e0117069. doi: 10.1371/journal.pone.0117069 (PMC4324630; doi:10.1371/journal.pone.0117069)
Supplement: S3 Table — (DOCX) [file pone.0117069.s003.docx]

**Supporting table S3- Variable sites in mitochondrial *D- loop region* sequences with respect to complete mitochondrial genome of red deer (GenBank accession no. NC-007704.2)**

| **Subspecies/Haplotypes** |  | **Variable positions** | | | | | | | | | | | | | | |  |
| --- | --- | --- | --- | --- | --- | --- | --- | --- | --- | --- | --- | --- | --- | --- | --- | --- | --- |
| **Nt. position** | **15500** | **15501** | **15503** | **15513** | **15526** | **15533** | **15535** | **15539** | **15547** | **15549** | **15550** | **15552** | **15553** | **15554** | **15555** | **15556** | **15557** |
| NC_007704.2 | G | T | T | C | C | C | C | T | T | T | ― | C | A | T | C | ― | C |
| *C.e. hanglu*/Hap-01 | ― | T | T | C | T | C | T | T | T | T | T | C | A | T | C | A | C |
| *C.e. hanglu* /Hap-02 | A | T | T | C | T | C | T | T | T | T | T | C | A | T | C | A | C |
| *C.e. hanglu* /Hap-03 | A | T | T | C | T | C | T | T | T | T | T | C | A | T | C | A | C |
| *C.e. hanglu* /Hap-04 | A | T | T | C | T | C | T | T | T | T | T | C | A | T | C | A | C |
| *C.e. hanglu* /Hap-05 | A | T | T | C | T | C | T | T | T | T | T | C | A | T | C | A | C |
| *C.e. hanglu* /Hap-06 | A | T | T | C | T | C | T | T | T | T | T | C | A | T | C | A | C |
| *C.e. hanglu* /Hap-07 | A | T | T | C | T | C | T | T | T | T | T | C | A | T | C | A | C |
| *C.e. hanglu* /Hap-08 | A | T | T | C | T | C | T | T | T | T | T | C | A | T | C | A | C |
| *C.e. hanglu* /Hap-09 | A | T | T | C | T | C | T | T | T | T | T | C | A | T | C | A | C |
| *C.e. hanglu* /Hap-10 | A | T | T | C | T | C | T | T | T | T | T | C | A | T | C | A | C |
| *C.e. hanglu* /Hap-11 | A | T | T | C | T | C | T | T | T | T | T | C | A | T | C | A | C |
| *C.e. hanglu* /Hap-12 | A | T | T | C | T | C | T | T | T | T | T | C | A | T | C | A | C |
| *C.e. hanglu* /Hap-13 | A | T | T | C | T | C | T | T | T | T | T | C | A | T | C | A | C |
| *C. e. yarkandensis* /Hap-14 | A | T | T | C | T | C | T | T | C | T | ― | T | A | T | T | A | C |
| *C. e. bactrianus* /Hap-15 | A | T | T | C | T | C | T | T | C | T | ― | T | A | T | T | A | C |
| *C. e. hippelaphus* /Hap-16 | G | T | T | C | C | C | T | T | T | T | T | C | A | T | C | ― | C |
| *C. e. hippelaphus* /Hap-17 | G | T | T | C | C | C | T | T | T | T | ― | C | A | T | C | ― | C |
| *C. e. hispanicus* /Hap-18 | G | T | T | C | C | C | C | T | T | T | ― | C | A | T | C | ― | C |
| *C. elaphus* /Hap-19 | G | T | T | C | C | C | C | T | T | T | ― | C | A | T | C | ― | C |
| *C.e. atlanticus* /Hap-20 | G | T | T | C | C | C | C | T | T | T | ― | C | A | T | C | ― | C |
| *C. e. barbarus* /Hap-21 | G | T | T | C | C | T | T | T | T | T | T | C | A | C | C | ― | C |
| *C.e. corsicanus* /Hap-22 | G | T | T | C | C | T | C | T | T | T | C | C | A | C | C | ― | C |
| *C. e. songaricus* /Hap-23 | A | ― | T | C | T | C | T | A | T | T | T | C | A | T | C | C | T |
| *C.e. kansuensis* /Hap-24 | A | ― | T | T | T | C | T | A | T | T | T | C | A | T | C | C | T |
| *C. e. wallichi* /Hap-25 | A | ― | T | T | T | C | T | A | T | T | ― | C | A | T | C | C | T |
| *C.e.manitobensis* /Hap-26 | A | A | T | T | T | C | T | A | T | T | ― | C | G | T | C | C | T |
| *C.e.nelsoni* /Hap-27 | A | ― | T | T | T | C | T | A | T | C | ― | C | G | T | C | C | T |
| *C.e.roosevelti* /Hap-28 | A | ― | T | T | T | C | T | A | T | T | ― | C | G | T | C | C | T |
| *C.e. xanthopygus* Hap-29 | A | A | T | T | T | C | T | A | T | T | T | C | A | T | C | C | T |
| *C. e. alashanicus* /Hap-30 | A | ― | T | T | T | C | T | A | T | T | ― | C | G | T | C | C | T |
| *C.e. sibiricus/*Hap-31 | A | A | C | T | T | C | T | A | T | T | ― | C | G | T | C | C | T |
| *C. e. canadensis* /Hap-32 | A | ― | T | T | T | C | T | A | T | T | ― | C | G | T | C | C | T |

Continued…

| **Subspecies/Haplotypes** | **Variable positions** | | | | | | | | | | | | | | | |  |
| --- | --- | --- | --- | --- | --- | --- | --- | --- | --- | --- | --- | --- | --- | --- | --- | --- | --- |
| **Nt. position** | **15560** | **15561** | **15563** | **15564** | **15566** | **15568** | **15570** | **15572** | **15573** | **15575** | **15576** | **15578** | **15579** | **15581** | **15583** | **15584** | **15588** |
| NC_007704.2 | C | T | C | C | C | C | A | A | A | A | T | T | G | A | T | A | A |
| *C.e. hanglu*/Hap-01 | T | A | C | C | C | T | A | A | A | G | C | C | A | A | T | A | T |
| *C.e. hanglu* /Hap-02 | T | A | C | C | C | T | A | A | A | G | C | C | A | A | T | A | T |
| *C.e. hanglu* /Hap-03 | T | A | C | C | C | T | A | A | A | G | C | C | A | A | T | A | T |
| *C.e. hanglu* /Hap-04 | T | A | C | C | C | T | A | A | A | G | C | G | A | A | A | A | T |
| *C.e. hanglu* /Hap-05 | T | A | C | C | C | T | A | A | A | G | C | G | A | A | A | A | T |
| *C.e. hanglu* /Hap-06 | T | A | C | C | C | T | A | A | A | G | C | G | A | A | T | A | T |
| *C.e. hanglu* /Hap-07 | T | A | C | C | C | T | A | A | A | G | C | G | A | A | A | A | T |
| *C.e. hanglu* /Hap-08 | T | A | C | C | C | T | A | A | A | G | C | G | A | A | A | A | T |
| *C.e. hanglu* /Hap-09 | T | A | C | C | C | T | A | A | A | G | C | G | A | A | T | A | T |
| *C.e. hanglu* /Hap-10 | T | A | C | C | C | T | A | A | A | G | C | G | A | A | T | A | T |
| *C.e. hanglu* /Hap-11 | T | A | C | C | C | T | A | A | A | G | C | G | A | A | T | A | T |
| *C.e. hanglu* /Hap-12 | T | A | C | C | C | T | A | A | A | G | C | G | A | A | A | A | T |
| *C.e. hanglu* /Hap-13 | T | A | C | C | C | T | A | A | A | G | C | G | A | A | A | A | T |
| *C. e. yarkandensis* /Hap-14 | T | A | C | C | C | T | G | A | A | A | C | C | A | A | T | A | T |
| *C. e. bactrianus* /Hap-15 | T | A | C | C | C | T | A | A | A | A | C | C | A | A | T | A | T |
| *C. e. hippelaphus* /Hap-16 | T | A | C | C | C | C | A | A | A | A | T | T | G | A | T | G | A |
| *C. e. hippelaphus* /Hap-17 | T | A | C | C | C | C | A | A | A | A | T | T | G | A | T | A | A |
| *C. e. hispanicus* /Hap-18 | C | A | C | C | C | C | A | A | A | A | T | T | G | A | T | A | A |
| *C. elaphus* /Hap-19 | C | A | C | C | C | C | A | A | A | A | T | T | G | A | T | A | A |
| *C.e. atlanticus* /Hap-20 | C | A | C | C | C | C | A | A | A | A | T | T | G | A | T | A | A |
| *C. e. barbarus* /Hap-21 | T | A | C | C | T | C | A | A | G | A | T | T | G | A | T | A | A |
| *C.e. corsicanus* /Hap-22 | T | A | C | C | T | C | A | A | G | A | T | T | G | A | T | A | A |
| *C. e. songaricus* /Hap-23 | T | A | T | T | C | C | G | A | A | A | C | T | A | G | T | A | T |
| *C.e. kansuensis* /Hap-24 | T | A | T | T | C | C | G | A | A | A | C | T | G | G | T | A | T |
| *C. e. wallichi* /Hap-25 | T | A | T | T | C | C | G | A | A | A | C | T | G | G | T | A | T |
| *C.e.manitobensis* /Hap-26 | T | A | T | T | C | C | G | A | A | A | C | T | G | G | T | A | T |
| *C.e.nelsoni* /Hap-27 | T | A | T | T | C | C | G | A | A | A | C | T | G | G | T | A | T |
| *C.e.roosevelti* /Hap-28 | T | A | T | T | C | C | G | G | A | A | C | T | G | G | T | A | T |
| *C.e.xanthopygus* Hap-29 | T | A | T | T | C | C | G | A | A | A | C | T | G | G | T | A | T |
| *C. e. alashanicus* /Hap-30 | T | A | T | T | C | C | G | A | A | A | C | T | G | G | T | A | T |
| *C.e. sibiricus/*Hap-31 | T | A | T | T | C | C | G | A | A | A | C | T | G | G | T | A | T |
| *C. e. canadensis* /Hap-32 | T | A | T | T | C | C | G | A | A | A | C | T | G | G | T | A | T |

Continued…

| **Subspecies/Haplotypes** | **Variable position** | | | | | | | | | | | | | | | |  |
| --- | --- | --- | --- | --- | --- | --- | --- | --- | --- | --- | --- | --- | --- | --- | --- | --- | --- |
| **Nt position** | **15591** | **15592** | **15593** | **15602** | **15603** | **15613** | **15616** | **15617** | **15618** | **15619** | **15620** | **15621** | **15622** | **15623** | **15624** | **15625** | **15626** |
| NC_007704.2 | C | C | T | T | A | G | T | ― | ― | ― | ― | ― | ― | ― | ― | ― | ― |
| *C.e. hanglu*/Hap-01 | C | C | C | T | A | G | T | ― | ― | ― | ― | ― | ― | ― | ― | ― | ― |
| *C.e. hanglu* /Hap-02 | C | C | C | T | A | G | T | ― | ― | ― | ― | ― | ― | ― | ― | ― | ― |
| *C.e. hanglu* /Hap-03 | C | C | C | T | A | G | T | ― | ― | ― | ― | ― | ― | ― | ― | ― | ― |
| *C.e. hanglu* /Hap-04 | C | C | C | T | A | G | T | ― | ― | ― | ― | ― | ― | ― | ― | ― | ― |
| *C.e. hanglu* /Hap-05 | C | C | C | T | A | G | T | ― | ― | ― | ― | ― | ― | ― | ― | ― | ― |
| *C.e. hanglu* /Hap-06 | C | C | C | T | A | G | T | ― | ― | ― | ― | ― | ― | ― | ― | ― | ― |
| *C.e. hanglu* /Hap-07 | C | C | C | T | A | G | G | ― | ― | ― | ― | ― | ― | ― | ― | ― | ― |
| *C.e. hanglu* /Hap-08 | C | C | C | T | A | G | G | ― | ―  119 bp deletion in Western and Tarim Red deer | ― | ― | ― | ― | ― | ― | ― | ― |
| *C.e. hanglu* /Hap-09 | C | C | C | T | A | G | T | ― | ― | ― | ― | ― | ― | ― | ― | ― | ― |
| *C.e. hanglu* /Hap-10 | C | C | C | T | A | G | T | ― | ― | ― | ― | ― | ― | ― | ― | ― | ― |
| *C.e. hanglu* /Hap-11 | C | C | C | T | A | G | T | ― | ― | ― | ― | ― | ― | ― | ― | ― | ― |
| *C.e. hanglu* /Hap-12 | C | C | C | T | A | G | T | ― | ― | ― | ― | ― | ― | ― | ― | ― | ― |
| *C.e. hanglu* /Hap-13 | C | C | C | T | A | G | G | ― | ― | ― | ― | ― | ― | ― | ― | ― | ― |
| *C. e. yarkandensis* /Hap-14 | C | C | C | T | A | G | T | ― | ― | ― | ― | ― | ― | ― | ― | ― | ― |
| *C. e. bactrianus* /Hap-15 | C | C | C | T | A | G | T | ― | ― | ― | ― | ― | ― | ― | ― | ― | ― |
| *C. e. hippelaphus* /Hap-16 | T | C | T | T | A | G | T | ― | ― | ― | ― | ― | ― | ― | ― | ― | ― |
| *C. e. hippelaphus* /Hap-17 | C | C | T | T | A | G | T | ― | ― | ― | ― | ― | ― | ― | ― | ― | ― |
| *C. e. hispanicus* /Hap-18 | C | C | T | T | A | A | T | ― | ― | ― | ― | ― | ― | ― | ― | ― | ― |
| *C. elaphus* /Hap-19 | C | C | T | T | A | G | T | ― | ― | ― | ― | ― | ― | ― | ― | ― | ― |
| *C.e. atlanticus* /Hap-20 | C | C | T | T | A | G | T | ― | ― | ― | ― | ― | ― | ― | ― | ― | ― |
| *C. e. barbarus* /Hap-21 | C | C | C | T | A | G | T | ― | ― | ― | ― | ― | ― | ― | ― | ― | ― |
| *C.e. corsicanus* /Hap-22 | C | T | T | T | A | A | T | ― | ― | ― | ― | ― | ― | ― | ― | ― | ― |
| *C. e. songaricus* /Hap-23 | C | C | T | C | G | A | T | C | A | A | T | G | T | G | C | T | A |
| *C.e. kansuensis* /Hap-24 | C | C | T | T | G | A | T | C | A | A | T | G | T | G | C | T | A |
| *C. e. wallichi* /Hap-25 | C | C | T | T | G | A | T | C | A | A | T | G | T | G | C | T | A |
| *C.e.manitobensis* /Hap-26 | C | C | T | C | G | A | T | C | A | A | T | G | T | G | C | T | A |
| *C.e.nelsoni* /Hap-27 | C | C | T | C | G | A | T | C | A | A | T | G | T | G | C | T | A |
| *C.e.roosevelti* /Hap-28 | C | C | T | C | G | A | T | C | A | A | T | G | T | G | C | T | A |
| *C.e.xanthopygus* Hap-29 | C | C | T | T | G | A | T | C | A | A | T | G | T | A | C | T | A |
| *C. e. alashanicus* /Hap-30 | C | C | T | T | G | A | T | C | A | A | T | G | T | G | C | T | A |
| *C.e. sibiricus/*Hap-31 | C | C | T | T | G | A | T | C | A | A | T | G | T | G | C | T | A |
| *C. e. canadensis* /Hap-32 | C | C | T | C | G | A | T | C | A | A | T | G | T | G | C | T | A |

Continued…

| **Subspecies/Haplotypes** | **Variable position** | | | | | | | | | | | | | | | |  |
| --- | --- | --- | --- | --- | --- | --- | --- | --- | --- | --- | --- | --- | --- | --- | --- | --- | --- |
| **Nt position** | **15627** | **15628** | **15629** | **15630** | **15631** | **15632** | **15633** | **15634** | **15635** | **15636** | **15637** | **15638** | **15639** | **15640** | **15641** | **15642** | **15643** |
| NC_007704.2 | ― | ― | ― | ― | ― | ― | ― | ― | ― | ― | ― | ― | ― | ― | ― | ― | ― |
| *C.e. hanglu*/Hap-01 | ― | ― | ― | ― | ― | ― | ― | ― | ― | ― | ― | ― | ― | ― | ― | ― | ― |
| *C.e. hanglu* /Hap-02 | ― | ― | ― | ― | ― | ― | ― | ― | ― | ― | ― | ― | ― | ― | ― | ― | ― |
| *C.e. hanglu* /Hap-03 | ― | ― | ― | ― | ― | ― | ― | ― | ― | ― | ― | ― | ― | ― | ― | ― | ― |
| *C.e. hanglu* /Hap-04 | ― | ― | ― | ― | ― | ― | ― | ― | ― | ― | ― | ― | ― | ― | ― | ― | ― |
| *C.e. hanglu* /Hap-05 | ― | ― | ― | ― | ― | ― | ― | ― | ― | ― | ― | ― | ― | ― | ― | ― | ― |
| *C.e. hanglu* /Hap-06 | ― | ― | ― | ― | ― | ― | ― | ― | ― | ― | ― | ― | ― | ― | ― | ― | ― |
| *C.e. hanglu* /Hap-07 | ― | ― | ― | ― | ― | ― | ―  119 bp deletion in Western and Tarim Red deer | ― | ― | ― | ― | ― | ― | ― | ― | ― | ― |
| *C.e. hanglu* /Hap-08 | ― | ― | ― | ― | ― | ― | ― | ― | ― | ― | ― | ― | ― | ― | ― | ― | ― |
| *C.e. hanglu* /Hap-09 | ― | ― | ― | ― | ― | ― | ― | ― | ― | ― | ― | ― | ― | ― | ― | ― | ― |
| *C.e. hanglu* /Hap-10 | ― | ― | ― | ― | ― | ― | ― | ― | ― | ― | ― | ― | ― | ― | ― | ― | ― |
| *C.e. hanglu* /Hap-11 | ― | ― | ― | ― | ― | ― | ― | ― | ― | ― | ― | ― | ― | ― | ― | ― | ― |
| *C.e. hanglu* /Hap-12 | ― | ― | ― | ― | ― | ― | ― | ― | ― | ― | ― | ― | ― | ― | ― | ― | ― |
| *C.e. hanglu* /Hap-13 | ― | ― | ― | ― | ― | ― | ― | ― | ― | ― | ― | ― | ― | ― | ― | ― | ― |
| *C. e. yarkandensis* /Hap-14 | ― | ― | ― | ― | ― | ― | ― | ― | ― | ― | ― | ― | ― | ― | ― | ― | ― |
| *C. e. bactrianus* /Hap-15 | ― | ― | ― | ― | ― | ― | ― | ― | ― | ― | ― | ― | ― | ― | ― | ― | ― |
| *C. e. hippelaphus* /Hap-16 | ― | ― | ― | ― | ― | ― | ― | ― | ― | ― | ― | ― | ― | ― | ― | ― | ― |
| *C. e. hippelaphus* /Hap-17 | ― | ― | ― | ― | ― | ― | ― | ― | ― | ― | ― | ― | ― | ― | ― | ― | ― |
| *C. e. hispanicus* /Hap-18 | ― | ― | ― | ― | ― | ― | ― | ― | ― | ― | ― | ― | ― | ― | ― | ― | ― |
| *C. elaphus* /Hap-19 | ― | ― | ― | ― | ― | ― | ― | ― | ― | ― | ― | ― | ― | ― | ― | ― | ― |
| *C.e. atlanticus* /Hap-20 | ― | ― | ― | ― | ― | ― | ― | ― | ― | ― | ― | ― | ― | ― | ― | ― | ― |
| *C. e. barbarus* /Hap-21 | ― | ― | ― | ― | ― | ― | ― | ― | ― | ― | ― | ― | ― | ― | ― | ― | ― |
| *C.e. corsicanus* /Hap-22 | ― | ― | ― | ― | ― | ― | ― | ― | ― | ― | ― | ― | ― | ― | ― | ― | ― |
| *C. e. songaricus* /Hap-23 | G | G | A | C | A | T | G | C | A | T | G | T | A | T | A | A | C |
| *C.e. kansuensis* /Hap-24 | G | G | A | C | G | T | G | C | A | T | G | T | A | T | A | A | C |
| *C. e. wallichi* /Hap-25 | G | G | A | C | G | T | G | C | A | T | G | T | A | T | A | A | C |
| *C.e.manitobensis* /Hap-26 | G | G | A | C | A | T | G | C | A | T | G | T | A | T | A | A | C |
| *C.e.nelsoni* /Hap-27 | G | G | A | C | A | T | G | C | A | T | G | T | A | T | A | A | C |
| *C.e.roosevelti* /Hap-28 | G | G | A | C | A | T | G | C | A | T | G | T | A | T | A | A | C |
| *C.e.xanthopygus* Hap-29 | G | G | A | C | A | T | G | C | A | T | G | T | A | T | A | A | C |
| *C. e. alashanicus* /Hap-30 | G | G | A | C | G | T | G | C | A | T | G | T | A | T | A | A | C |
| *C.e. sibiricus/*Hap-31 | G | A | A | C | A | C | G | C | A | T | G | T | A | T | A | A | C |
| *C. e. canadensis* /Hap-32 | G | G | A | C | A | T | G | C | A | T | G | T | A | T | A | A | C |

Continued…

| **Subspecies/Haplotypes** | **Variable position** | | | | | | | | | | | | | | | |  |
| --- | --- | --- | --- | --- | --- | --- | --- | --- | --- | --- | --- | --- | --- | --- | --- | --- | --- |
| **Nt. position** | **15644** | **15645** | **15646** | **15647** | **15648** | **15649** | **15650** | **15651** | **15652** | **15653** | **15654** | **15655** | **15656** | **15657** | **15658** | **15659** | **15660** |
| NC_007704.2 | ― | ― | ― | ― | ― | ― | ― | ― | ― | ― | ― | ― | ― | ― | ― | ― | ― |
| *C.e. hanglu*/Hap-01 | ― | ― | ― | ― | ― | ― | ― | ― | ― | ― | ― | ― | ― | ― | ― | ― | ― |
| *C.e. hanglu* /Hap-02 | ― | ― | ― | ― | ― | ― | ― | ― | ― | ― | ― | ― | ― | ― | ― | ― | ― |
| *C.e. hanglu* /Hap-03 | ― | ― | ― | ― | ― | ― | ― | ― | ― | ― | ― | ― | ― | ― | ― | ― | ― |
| *C.e. hanglu* /Hap-04 | ― | ― | ― | ― | ― | ― | ― | ― | ― | ― | ― | ― | ― | ― | ― | ― | ― |
| *C.e. hanglu* /Hap-05 | ― | ― | ― | ― | ― | ― | ― | ― | ― | ― | ― | ― | ― | ― | ― | ― | ― |
| *C.e. hanglu* /Hap-06 | ― | ― | ― | ― | ― | ― | ―  119 bp deletion in Western and Tarim Red deer | ― | ― | ― | ― | ― | ― | ― | ― | ― | ― |
| *C.e. hanglu* /Hap-07 | ― | ― | ― | ― | ― | ― | ― | ― | ― | ― | ― | ― | ― | ― | ― | ― | ― |
| *C.e. hanglu* /Hap-08 | ― | ― | ― | ― | ― | ― | ― | ― | ― | ― | ― | ― | ― | ― | ― | ― | ― |
| *C.e. hanglu* /Hap-09 | ― | ― | ― | ― | ― | ― | ― | ― | ― | ― | ― | ― | ― | ― | ― | ― | ― |
| *C.e. hanglu* /Hap-10 | ― | ― | ― | ― | ― | ― | ― | ― | ― | ― | ― | ― | ― | ― | ― | ― | ― |
| *C.e. hanglu* /Hap-11 | ― | ― | ― | ― | ― | ― | ― | ― | ― | ― | ― | ― | ― | ― | ― | ― | ― |
| *C.e. hanglu* /Hap-12 | ― | ― | ― | ― | ― | ― | ― | ― | ― | ― | ― | ― | ― | ― | ― | ― | ― |
| *C.e. hanglu* /Hap-13 | ― | ― | ― | ― | ― | ― | ― | ― | ― | ― | ― | ― | ― | ― | ― | ― | ― |
| *C. e. yarkandensis* /Hap-14 | ― | ― | ― | ― | ― | ― | ― | ― | ― | ― | ― | ― | ― | ― | ― | ― | ― |
| *C. e. bactrianus* /Hap-15 | ― | ― | ― | ― | ― | ― | ― | ― | ― | ― | ― | ― | ― | ― | ― | ― | ― |
| *C. e. hippelaphus* /Hap-16 | ― | ― | ― | ― | ― | ― | ― | ― | ― | ― | ― | ― | ― | ― | ― | ― | ― |
| *C. e. hippelaphus* /Hap-17 | ― | ― | ― | ― | ― | ― | ― | ― | ― | ― | ― | ― | ― | ― | ― | ― | ― |
| *C. e. hispanicus* /Hap-18 | ― | ― | ― | ― | ― | ― | ― | ― | ― | ― | ― | ― | ― | ― | ― | ― | ― |
| *C. elaphus* /Hap-19 | ― | ― | ― | ― | ― | ― | ― | ― | ― | ― | ― | ― | ― | ― | ― | ― | ― |
| *C.e. atlanticus* /Hap-20 | ― | ― | ― | ― | ― | ― | ― | ― | ― | ― | ― | ― | ― | ― | ― | ― | ― |
| *C. e. barbarus* /Hap-21 | ― | ― | ― | ― | ― | ― | ― | ― | ― | ― | ― | ― | ― | ― | ― | ― | ― |
| *C.e. corsicanus* /Hap-22 | ― | ― | ― | ― | ― | ― | ― | ― | ― | ― | ― | ― | ― | ― | ― | ― | ― |
| *C. e. songaricus* /Hap-23 | A | G | T | A | C | A | T | G | A | G | T | T | A | G | C | G | T |
| *C.e. kansuensis* /Hap-24 | A | G | T | A | C | A | T | G | A | G | T | T | A | T | T | G | T |
| *C. e. wallichi* /Hap-25 | A | G | T | A | C | A | T | G | A | G | T | T | A | G | T | G | T |
| *C.e.manitobensis* /Hap-26 | A | G | T | A | C | A | T | G | A | G | T | T | A | G | C | G | T |
| *C.e.nelsoni* /Hap-27 | A | G | T | A | C | A | T | G | A | G | T | T | A | G | C | G | T |
| *C.e.roosevelti* /Hap-28 | A | G | T | A | C | A | T | G | A | G | T | T | A | G | C | G | T |
| *C.e.xanthopygus* Hap-29 | A | G | T | A | C | A | T | A | A | G | T | T | A | G | C | G | T |
| *C. e. alashanicus* /Hap-30 | A | G | T | A | C | A | T | G | A | G | T | T | G | G | T | G | T |
| *C.e. sibiricus/*Hap-31 | A | G | T | C | C | A | T | G | A | G | T | T | A | G | T | G | T |
| *C. e. canadensis* /Hap-32 | A | G | T | A | C | A | T | G | A | G | T | T | A | G | C | G | T |

Continued…

| **Subspecies/Haplotypes** | **Variable position** | | | | | | | | | | | | | | | |  |
| --- | --- | --- | --- | --- | --- | --- | --- | --- | --- | --- | --- | --- | --- | --- | --- | --- | --- |
| **Nt. position** | **15661** | **15662** | **15663** | **15664** | **15665** | **15666** | **15667** | **15668** | **15669** | **15670** | **15671** | **15672** | **15673** | **15674** | **15675** | **15676** | **15677** |
| NC_007704.2 | ― | ― | ― | ― | ― | ― | ― | ― | ― | ― | ― | ― | ― | ― | ― | ― | ― |
| *C.e. hanglu*/Hap-01 | ― | ― | ― | ― | ― | ― | ― | ― | ― | ― | ― | ― | ― | ― | ― | ― | ― |
| *C.e. hanglu* /Hap-02 | ― | ― | ― | ― | ― | ― | ― | ― | ― | ― | ― | ― | ― | ― | ― | ― | ― |
| *C.e. hanglu* /Hap-03 | ― | ― | ― | ― | ― | ― | ― | ― | ― | ― | ― | ― | ― | ― | ― | ― | ― |
| *C.e. hanglu* /Hap-04 | ― | ― | ― | ― | ― | ― | ― | ― | ― | ― | ― | ― | ― | ― | ― | ― | ― |
| *C.e. hanglu* /Hap-05 | ― | ― | ― | ― | ― | ― | ― | ― | ― | ― | ― | ― | ― | ― | ― | ― | ― |
| *C.e. hanglu* /Hap-06 | ― | ― | ― | ― | ― | ― | ― | ― | ― | ― | ― | ― | ― | ― | ― | ― | ― |
| *C.e. hanglu* /Hap-07 | ― | ― | ― | ― | ― | ―  119 bp deletion in Western and Tarim Red deer | ― | ― | ― | ― | ― | ― | ― | ― | ― | ― | ― |
| *C.e. hanglu* /Hap-08 | ― | ― | ― | ― | ― | ― | ― | ― | ― | ― | ― | ― | ― | ― | ― | ― | ― |
| *C.e. hanglu* /Hap-09 | ― | ― | ― | ― | ― | ― | ― | ― | ― | ― | ― | ― | ― | ― | ― | ― | ― |
| *C.e. hanglu* /Hap-10 | ― | ― | ― | ― | ― | ― | ― | ― | ― | ― | ― | ― | ― | ― | ― | ― | ― |
| *C.e. hanglu* /Hap-11 | ― | ― | ― | ― | ― | ― | ― | ― | ― | ― | ― | ― | ― | ― | ― | ― | ― |
| *C.e. hanglu* /Hap-12 | ― | ― | ― | ― | ― | ― | ― | ― | ― | ― | ― | ― | ― | ― | ― | ― | ― |
| *C.e. hanglu* /Hap-13 | ― | ― | ― | ― | ― | ― | ― | ― | ― | ― | ― | ― | ― | ― | ― | ― | ― |
| *C. e. yarkandensis* /Hap-14 | ― | ― | ― | ― | ― | ― | ― | ― | ― | ― | ― | ― | ― | ― | ― | ― | ― |
| *C. e. bactrianus* /Hap-15 | ― | ― | ― | ― | ― | ― | ― | ― | ― | ― | ― | ― | ― | ― | ― | ― | ― |
| *C. e. hippelaphus* /Hap-16 | ― | ― | ― | ― | ― | ― | ― | ― | ― | ― | ― | ― | ― | ― | ― | ― | ― |
| *C. e. hippelaphus* /Hap-17 | ― | ― | ― | ― | ― | ― | ― | ― | ― | ― | ― | ― | ― | ― | ― | ― | ― |
| *C. e. hispanicus* /Hap-18 | ― | ― | ― | ― | ― | ― | ― | ― | ― | ― | ― | ― | ― | ― | ― | ― | ― |
| *C. elaphus* /Hap-19 | ― | ― | ― | ― | ― | ― | ― | ― | ― | ― | ― | ― | ― | ― | ― | ― | ― |
| *C.e. atlanticus* /Hap-20 | ― | ― | ― | ― | ― | ― | ― | ― | ― | ― | ― | ― | ― | ― | ― | ― | ― |
| *C. e. barbarus* /Hap-21 | ― | ― | ― | ― | ― | ― | ― | ― | ― | ― | ― | ― | ― | ― | ― | ― | ― |
| *C.e. corsicanus* /Hap-22 | ― | ― | ― | ― | ― | ― | ― | ― | ― | ― | ― | ― | ― | ― | ― | ― | ― |
| *C. e. songaricus* /Hap-23 | A | T | A | G | G | A | C | A | T | A | T | T | A | T | G | C | A |
| *C.e. kansuensis* /Hap-24 | A | T | A | G | G | A | C | A | T | A | T | T | A | T | G | T | A |
| *C. e. wallichi* /Hap-25 | A | T | A | G | G | A | C | A | T | A | T | T | A | T | G | T | A |
| *C.e.manitobensis* /Hap-26 | A | T | A | G | G | A | C | A | T | A | T | T | A | T | G | T | A |
| *C.e.nelsoni* /Hap-27 | A | T | A | G | G | A | C | A | T | A | T | T | A | T | G | T | A |
| *C.e.roosevelti* /Hap-28 | A | T | A | G | G | A | C | A | T | A | T | T | A | T | G | T | A |
| *C.e.xanthopygus* Hap-29 | A | T | A | G | G | A | C | A | T | A | T | T | A | T | G | T | A |
| *C. e. alashanicus* /Hap-30 | A | T | A | G | G | A | C | A | T | A | T | T | A | T | G | T | A |
| *C.e. sibiricus/*Hap-31 | A | T | A | G | G | A | C | A | T | A | T | T | A | T | G | T | A |
| *C. e. canadensis* /Hap-32 | A | T | A | G | G | A | C | A | T | A | T | T | A | T | G | T | A |

| **Subspecies/Haplotypes** |  | **Variable position** | | | | | | | | | | | | | | |  |
| --- | --- | --- | --- | --- | --- | --- | --- | --- | --- | --- | --- | --- | --- | --- | --- | --- | --- |
| **Nt. position** | **15678** | **15679** | **15680** | **15681** | **15682** | **15683** | **15684** | **15685** | **15686** | **15687** | **15688** | **15689** | **15690** | **15691** | **15692** | **15693** | **15694** |
| NC_007704.2 | ― | ― | ― | ― | ― | ― | ― | ― | ― | ― | ― | ― | ― | ― | ― | ― | ― |
| *C.e. hanglu*/Hap-01 | ― | ― | ― | ― | ― | ― | ― | ― | ― | ― | ― | ― | ― | ― | ― | ― | ― |
| *C.e. hanglu* /Hap-02 | ― | ― | ― | ― | ― | ― | ― | ― | ― | ― | ― | ― | ― | ― | ― | ― | ― |
| *C.e. hanglu* /Hap-03 | ― | ― | ― | ― | ― | ― | ― | ― | ― | ― | ― | ― | ― | ― | ― | ― | ― |
| *C.e. hanglu* /Hap-04 | ― | ― | ― | ― | ― | ― | ― | ― | ― | ― | ― | ― | ― | ― | ― | ― | ― |
| *C.e. hanglu* /Hap-05 | ― | ― | ― | ― | ― | ― | ― | ― | ― | ― | ― | ― | ― | ― | ― | ― | ― |
| *C.e. hanglu* /Hap-06 | ― | ― | ― | ― | ―  119 bp deletion in Western and Tarim Red deer | ― | ― | ― | ― | ― | ― | ― | ― | ― | ― | ― | ― |
| *C.e. hanglu* /Hap-07 | ― | ― | ― | ― | ― | ― | ― | ― | ― | ― | ― | ― | ― | ― | ― | ― | ― |
| *C.e. hanglu* /Hap-08 | ― | ― | ― | ― | ― | ― | ― | ― | ― | ― | ― | ― | ― | ― | ― | ― | ― |
| *C.e. hanglu* /Hap-09 | ― | ― | ― | ― | ― | ― | ― | ― | ― | ― | ― | ― | ― | ― | ― | ― | ― |
| *C.e. hanglu* /Hap-10 | ― | ― | ― | ― | ― | ― | ― | ― | ― | ― | ― | ― | ― | ― | ― | ― | ― |
| *C.e. hanglu* /Hap-11 | ― | ― | ― | ― | ― | ― | ― | ― | ― | ― | ― | ― | ― | ― | ― | ― | ― |
| *C.e. hanglu* /Hap-12 | ― | ― | ― | ― | ― | ― | ― | ― | ― | ― | ― | ― | ― | ― | ― | ― | ― |
| *C.e. hanglu* /Hap-13 | ― | ― | ― | ― | ― | ― | ― | ― | ― | ― | ― | ― | ― | ― | ― | ― | ― |
| *C. e. yarkandensis* /Hap-14 | ― | ― | ― | ― | ― | ― | ― | ― | ― | ― | ― | ― | ― | ― | ― | ― | ― |
| *C. e. bactrianus* /Hap-15 | ― | ― | ― | ― | ― | ― | ― | ― | ― | ― | ― | ― | ― | ― | ― | ― | ― |
| *C. e. hippelaphus* /Hap-16 | ― | ― | ― | ― | ― | ― | ― | ― | ― | ― | ― | ― | ― | ― | ― | ― | ― |
| *C. e. hippelaphus* /Hap-17 | ― | ― | ― | ― | ― | ― | ― | ― | ― | ― | ― | ― | ― | ― | ― | ― | ― |
| *C. e. hispanicus* /Hap-18 | ― | ― | ― | ― | ― | ― | ― | ― | ― | ― | ― | ― | ― | ― | ― | ― | ― |
| *C. elaphus* /Hap-19 | ― | ― | ― | ― | ― | ― | ― | ― | ― | ― | ― | ― | ― | ― | ― | ― | ― |
| *C.e. atlanticus* /Hap-20 | ― | ― | ― | ― | ― | ― | ― | ― | ― | ― | ― | ― | ― | ― | ― | ― | ― |
| *C. e. barbarus* /Hap-21 | ― | ― | ― | ― | ― | ― | ― | ― | ― | ― | ― | ― | ― | ― | ― | ― | ― |
| *C.e. corsicanus* /Hap-22 | ― | ― | ― | ― | ― | ― | ― | ― | ― | ― | ― | ― | ― | ― | ― | ― | ― |
| *C. e. songaricus* /Hap-23 | T | A | A | T | A | G | T | A | C | A | T | ― | ― | ― | ― | ― | ― |
| *C.e. kansuensis* /Hap-24 | T | A | A | T | A | G | T | A | C | A | T | ― | ― | ― | ― | ― | ― |
| *C. e. wallichi* /Hap-25 | T | A | A | T | A | G | T | A | C | A | T | ―  44 bp deletion in Eastern Red deer | ― | ― | ― | ― | ― |
| *C.e.manitobensis* /Hap-26 | T | A | A | T | A | G | T | A | C | A | T | ― | ― | ― | ― | ― | ― |
| *C.e.nelsoni* /Hap-27 | T | A | A | T | A | G | T | A | C | A | T | ― | ― | ― | ― | ― | ― |
| *C.e.roosevelti* /Hap-28 | T | A | A | T | A | G | T | A | C | A | T | ― | ― | ― | ― | ― | ― |
| *C.e.xanthopygus* Hap-29 | T | A | A | T | A | G | T | A | C | A | T | ― | ― | ― | ― | ― | ― |
| *C. e. alashanicus* /Hap-30 | T | A | A | T | A | G | T | A | C | A | T | ― | ― | ― | ― | ― | ― |
| *C.e. sibiricus/*Hap-31 | T | A | A | T | A | G | T | A | C | A | T | ― | ― | ― | ― | ― | ― |
| *C. e. canadensis* /Hap-32 | T | A | A | T | A | G | T | A | C | A | T | ― | ― | ― | ― | ― | ― |

Continued…

| **Subspecies/Haplotypes** | **Variable position** | | | | | | | | | | | | | | | |  |
| --- | --- | --- | --- | --- | --- | --- | --- | --- | --- | --- | --- | --- | --- | --- | --- | --- | --- |
| **Nt. position** | **15695** | **15696** | **15697** | **15698** | **15699** | **15700** | **15701** | **15702** | **15703** | **15704** | **15705** | **15706** | **15707** | **15708** | **15709** | **15710** | **15711** |
| NC_007704.2 | ― | ― | ― | ― | ― | ― | ― | ― | ― | ― | ― | ― | ― | ― | ― | ― | ― |
| *C.e. hanglu*/Hap-01 | ― | ― | ― | ― | ― | ― | ― | ― | ― | ― | ― | ― | ― | ― | ― | ― | ― |
| *C.e. hanglu* /Hap-02 | ― | ― | ― | ― | ― | ― | ― | ― | ― | ― | ― | ― | ― | ― | ― | ― | ― |
| *C.e. hanglu* /Hap-03 | ― | ― | ― | ― | ― | ― | ― | ― | ― | ― | ― | ― | ― | ― | ― | ― | ― |
| *C.e. hanglu* /Hap-04 | ― | ― | ― | ― | ― | ― | ― | ― | ― | ― | ― | ― | ― | ― | ― | ― | ― |
| *C.e. hanglu* /Hap-05 | ― | ― | ― | ― | ― | ― | ― | ― | ― | ― | ― | ― | ― | ― | ― | ― | ― |
| *C.e. hanglu* /Hap-06 | ― | ― | ― | ― | ― | ― | ― | ― | ― | ― | ― | ― | ― | ― | ― | ― | ― |
| *C.e. hanglu* /Hap-07 | ― | ― | ― | ― | ― | ― | ― | ― | ― | ― | ― | ― | ― | ― | ― | ― | ― |
| *C.e. hanglu* /Hap-08 | ― | ― | ― | ― | ― | ―  119 bp deletion in Western and Tarim Red deer | ― | ― | ― | ― | ― | ― | ― | ― | ― | ― | ― |
| *C.e. hanglu* /Hap-09 | ― | ― | ― | ― | ― | ― | ― | ― | ― | ― | ― | ― | ― | ― | ― | ― | ― |
| *C.e. hanglu* /Hap-10 | ― | ― | ― | ― | ― | ― | ― | ― | ― | ― | ― | ― | ― | ― | ― | ― | ― |
| *C.e. hanglu* /Hap-11 | ― | ― | ― | ― | ― | ― | ― | ― | ― | ― | ― | ― | ― | ― | ― | ― | ― |
| *C.e. hanglu* /Hap-12 | ― | ― | ― | ― | ― | ― | ― | ― | ― | ― | ― | ― | ― | ― | ― | ― | ― |
| *C.e. hanglu* /Hap-13 | ― | ― | ― | ― | ― | ― | ― | ― | ― | ― | ― | ― | ― | ― | ― | ― | ― |
| *C. e. yarkandensis* /Hap-14 | ― | ― | ― | ― | ― | ― | ― | ― | ― | ― | ― | ― | ― | ― | ― | ― | ― |
| *C. e. bactrianus* /Hap-15 | ― | ― | ― | ― | ― | ― | ― | ― | ― | ― | ― | ― | ― | ― | ― | ― | ― |
| *C. e. hippelaphus* /Hap-16 | ― | ― | ― | ― | ― | ― | ― | ― | ― | ― | ― | ― | ― | ― | ― | ― | ― |
| *C. e. hippelaphus* /Hap-17 | ― | ― | ― | ― | ― | ― | ― | ― | ― | ― | ― | ― | ― | ― | ― | ― | ― |
| *C. e. hispanicus* /Hap-18 | ― | ― | ― | ― | ― | ― | ― | ― | ― | ― | ― | ― | ― | ― | ― | ― | ― |
| *C. elaphus* /Hap-19 | ― | ― | ― | ― | ― | ― | ― | ― | ― | ― | ― | ― | ― | ― | ― | ― | ― |
| *C.e. atlanticus* /Hap-20 | ― | ― | ― | ― | ― | ― | ― | ― | ― | ― | ― | ― | ― | ― | ― | ― | ― |
| *C. e. barbarus* /Hap-21 | ― | ― | ― | ― | ― | ― | ― | ― | ― | ― | ― | ― | ― | ― | ― | ― | ― |
| *C.e. corsicanus* /Hap-22 | ― | ― | ― | ― | ― | ― | ― | ― | ― | ― | ― | ― | ― | ― | ― | ― | ― |
| *C. e. songaricus* /Hap-23 | ― | ― | ― | ― | ― | ― | ― | ― | ― | ― | ― | ― | ― | ― | ― | ― | ― |
| *C.e. kansuensis* /Hap-24 | ― | ― | ― | ― | ― | ― | ― | ― | ― | ― | ― | ― | ― | ― | ― | ― | ― |
| *C. e. wallichi* /Hap-25 | ― | ― | ― | ― | ― | ― | ― | ― | ― | ― | ― | ― | ― | ― | ― | ― | ― |
| *C.e.manitobensis* /Hap-26 | ― | ― | ― | ― | ―  44 bp deletion in Eastern Red deer | ― | ― | ― | ― | ― | ― | ― | ― | ― | ― | ― | ― |
| *C.e.nelsoni* /Hap-27 | ― | ― | ― | ― | ― | ― | ― | ― | ― | ― | ― | ― | ― | ― | ― | ― | ― |
| *C.e.roosevelti* /Hap-28 | ― | ― | ― | ― | ― | ― | ― | ― | ― | ― | ― | ― | ― | ― | ― | ― | ― |
| *C.e.xanthopygus* Hap-29 | ― | ― | ― | ― | ― | ― | ― | ― | ― | ― | ― | ― | ― | ― | ― | ― | ― |
| *C. e. alashanicus* /Hap-30 | ― | ― | ― | ― | ― | ― | ― | ― | ― | ― | ― | ― | ― | ― | ― | ― | ― |
| *C.e. sibiricus/*Hap-31 | ― | ― | ― | ― | ― | ― | ― | ― | ― | ― | ― | ― | ― | ― | ― | ― | ― |
| *C. e. canadensis* /Hap-32 | ― | ― | ― | ― | ― | ― | ― | ― | ― | ― | ― | ― | ― | ― | ― | ― | ― |

Continued…

| **Subspecies/Haplotypes** | **Variable position** | | | | | | | | | | | | | | | |  |
| --- | --- | --- | --- | --- | --- | --- | --- | --- | --- | --- | --- | --- | --- | --- | --- | --- | --- |
| **Nt position** | **15712** | **15713** | **15714** | **15715** | **15716** | **15717** | **15718** | **15719** | **15720** | **15721** | **15722** | **15723** | **15724** | **15725** | **15726** | **15727** | **15728** |
| NC_007704.2 | ― | ― | ― | ― | ― | ― | ― | ― | ― | ― | ― | ― | ― | ― | ― | ― | ― |
| *C.e. hanglu*/Hap-01 | ― | ― | ― | ― | ― | ― | ― | ― | ― | ― | ― | ― | ― | ― | ― | ― | ― |
| *C.e. hanglu* /Hap-02 | ― | ― | ― | ― | ― | ― | ― | ― | ― | ― | ― | ― | ― | ― | ― | ― | ― |
| *C.e. hanglu* /Hap-03 | ― | ― | ― | ― | ― | ― | ― | ― | ― | ― | ― | ― | ― | ― | ― | ― | ― |
| *C.e. hanglu* /Hap-04 | ― | ― | ― | ― | ― | ― | ― | ― | ― | ― | ― | ― | ― | ― | ― | ― | ― |
| *C.e. hanglu* /Hap-05 | ― | ― | ― | ― | ― | ― | ― | ― | ― | ― | ― | ― | ― | ― | ― | ― | ― |
| *C.e. hanglu* /Hap-06 | ― | ― | ― | ― | ― | ― | ― | ― | ― | ― | ― | ― | ― | ― | ― | ― | ― |
| *C.e. hanglu* /Hap-07 | ― | ― | ― | ― | ― | ― | ― | ― | ― | ― | ― | ― | ― | ― | ― | ― | ― |
| *C.e. hanglu* /Hap-08 | ― | ― | ― | ― | ― | ―  119 bp deletion in Western and Tarim Red deer | ― | ― | ― | ― | ― | ― | ― | ― | ― | ― | ― |
| *C.e. hanglu* /Hap-09 | ― | ― | ― | ― | ― | ― | ― | ― | ― | ― | ― | ― | ― | ― | ― | ― | ― |
| *C.e. hanglu* /Hap-10 | ― | ― | ― | ― | ― | ― | ― | ― | ― | ― | ― | ― | ― | ― | ― | ― | ― |
| *C.e. hanglu* /Hap-11 | ― | ― | ― | ― | ― | ― | ― | ― | ― | ― | ― | ― | ― | ― | ― | ― | ― |
| *C.e. hanglu* /Hap-12 | ― | ― | ― | ― | ― | ― | ― | ― | ― | ― | ― | ― | ― | ― | ― | ― | ― |
| *C.e. hanglu* /Hap-13 | ― | ― | ― | ― | ― | ― | ― | ― | ― | ― | ― | ― | ― | ― | ― | ― | ― |
| *C. e. yarkandensis* /Hap-14 | ― | ― | ― | ― | ― | ― | ― | ― | ― | ― | ― | ― | ― | ― | ― | ― | ― |
| *C. e. bactrianus* /Hap-15 | ― | ― | ― | ― | ― | ― | ― | ― | ― | ― | ― | ― | ― | ― | ― | ― | ― |
| *C. e. hippelaphus* /Hap-16 | ― | ― | ― | ― | ― | ― | ― | ― | ― | ― | ― | ― | ― | ― | ― | ― | ― |
| *C. e. hippelaphus* /Hap-17 | ― | ― | ― | ― | ― | ― | ― | ― | ― | ― | ― | ― | ― | ― | ― | ― | ― |
| *C. e. hispanicus* /Hap-18 | ― | ― | ― | ― | ― | ― | ― | ― | ― | ― | ― | ― | ― | ― | ― | ― | ― |
| *C. elaphus* /Hap-19 | ― | ― | ― | ― | ― | ― | ― | ― | ― | ― | ― | ― | ― | ― | ― | ― | ― |
| *C.e. atlanticus* /Hap-20 | ― | ― | ― | ― | ― | ― | ― | ― | ― | ― | ― | ― | ― | ― | ― | ― | ― |
| *C. e. barbarus* /Hap-21 | ― | ― | ― | ― | ― | ― | ― | ― | ― | ― | ― | ― | ― | ― | ― | ― | ― |
| *C.e. corsicanus* /Hap-22 | ― | ― | ― | ― | ― | ― | ― | ― | ― | ― | ― | ― | ― | ― | ― | ― | ― |
| *C. e. songaricus* /Hap-23 | ― | ― | ― | ― | ― | ― | ― | ― | ― | ― | ― | ― | ― | ― | ― | ― | ― |
| *C.e. kansuensis* /Hap-24 | ― | ― | ― | ― | ― | ― | ― | ― | ― | ― | ― | ― | ― | ― | ― | ― | ― |
| *C. e. wallichi* /Hap-25 | ― | ― | ― | ― | ― | ―  44 bp deletion in Eastern Red deer | ― | ― | ― | ― | ― | ― | ― | ― | ― | ― | ― |
| *C.e.manitobensis* /Hap-26 | ― | ― | ― | ― | ― | ― | ― | ― | ― | ― | ― | ― | ― | ― | ― | ― | ― |
| *C.e.nelsoni* /Hap-27 | ― | ― | ― | ― | ― | ― | ― | ― | ― | ― | ― | ― | ― | ― | ― | ― | ― |
| *C.e.roosevelti* /Hap-28 | ― | ― | ― | ― | ― | ― | ― | ― | ― | ― | ― | ― | ― | ― | ― | ― | ― |
| *C.e.xanthopygus* Hap-29 | ― | ― | ― | ― | ― | ― | ― | ― | ― | ― | ― | ― | ― | ― | ― | ― | ― |
| *C. e. alashanicus* /Hap-30 | ― | ― | ― | ― | ― | ― | ― | ― | ― | ― | ― | ― | ― | ― | ― | ― | ― |
| *C.e. sibiricus/*Hap-31 | ― | ― | ― | ― | ― | ― | ― | ― | ― | ― | ― | ― | ― | ― | ― | ― | ― |
| *C. e. canadensis* /Hap-32 | ― | ― | ― | ― | ― | ― | ― | ― | ― | ― | ― | ― | ― | ― | ― | ― | ― |

Continued…

| **Subspecies/Haplotypes** |  | **Variable position** | | | | | | | | | | | | | | |  |  |
| --- | --- | --- | --- | --- | --- | --- | --- | --- | --- | --- | --- | --- | --- | --- | --- | --- | --- | --- |
| **Nt position** | **15729** | **15730** | **15731** | **15732** | **15733** | **15734** | **15735** | **15736** | **15737** | **15744** | **15745** | **15747** | **15754** | **15792** | **15805** | **15807** | **15808** | |
|  |  |  |  |  |  |  |  |  |  |  |  |  |  |  |  |  |  | |
| NC_007704.2 | ― | ― | ― | ― | ― | ― | ― | ― | ― | C | T | G | C | A | C | T | T | |
| *C.e. hanglu*/Hap-01 | ― | ― | ― | ― | ― | ― | ― | ― | ― | T | C | G | C | T | C | C | T | |
| *C.e. hanglu* /Hap-02 | ― | ― | ― | ― | ― | ― | ― | ― | ― | T | C | G | C | T | C | C | T | |
| *C.e. hanglu* /Hap-03 | ― | ― | ― | ― | ― | ― | ― | ― | ― | T | C | G | C | T | C | C | T | |
| *C.e. hanglu* /Hap-04 | ― | ― | ― | ― | ― | ― | ― | ― | ― | T | C | G | C | T | C | C | T | |
| *C.e. hanglu* /Hap-05 | ― | ― | ― | ― | ― | ― | ― | ― | ― | T | C | G | C | T | C | C | T | |
| *C.e. hanglu* /Hap-06 | ― | ― | ― | ― | ― | ― | ― | ― | ― | T | C | G | C | T | C | C | T | |
| *C.e. hanglu* /Hap-07 | ― | ― | ― | ― | ― | ― | ― | ― | ― | T | C | G | C | T | C | C | T | |
| *C.e. hanglu* /Hap-08 | ― | ― | ― | ― | ― | ― | ― | ― | ― | T | C | G | C | T | C | C | T | |
| *C.e. hanglu* /Hap-09 | ― | ― | ― | ― | ― | ― | ― | ― | ― | T | C | G | C | T | C | C | T | |
| *C.e. hanglu* /Hap-10 | ―  119 bp deletion in Western and Tarim Red deer | ― | ― | ― | ― | ― | ― | ― | ― | T | C | G | C | T | C | C | T | |
| *C.e. hanglu* /Hap-11 | ― | ― | ― | ― | ― | ― | ― | ― | ― | T | C | G | C | T | C | C | T | |
| *C.e. hanglu* /Hap-12 | ― | ― | ― | ― | ― | ― | ― | ― | ― | T | C | G | C | T | C | C | T | |
| *C.e. hanglu* /Hap-13 | ― | ― | ― | ― | ― | ― | ― | ― | ― | T | C | G | C | T | C | C | T | |
| *C. e. yarkandensis* /Hap-14 | ― | ― | ― | ― | ― | ― | ― | ― | ― | T | T | G | C | T | C | T | T | |
| *C. e. bactrianus* /Hap-15 | ― | ― | ― | ― | ― | ― | ― | ― | ― | T | T | G | C | T | C | T | T | |
| *C. e. hippelaphus* /Hap-16 | ― | ― | ― | ― | ― | ― | ― | ― | ― | C | T | G | C | T | C | T | T | |
| *C. e. hippelaphus* /Hap-17 | ― | ― | ― | ― | ― | ― | ― | ― | ― | C | T | G | C | T | T | T | T | |
| *C. e. hispanicus* /Hap-18 | ― | ― | ― | ― | ― | ― | ― | ― | ― | C | T | G | C | A | C | T | T | |
| *C. elaphus* /Hap-19 | ― | ― | ― | ― | ― | ― | ― | ― | ― | C | T | G | T | A | C | T | T | |
| *C.e. atlanticus* /Hap-20 | ― | ― | ― | ― | ― | ― | ― | ― | ― | C | T | G | C | A | T | T | T | |
| *C. e. barbarus* /Hap-21 | ― | ― | ― | ― | ― | ― | ― | ― | ― | T | T | G | T | T | T | T | T | |
| *C.e. corsicanus* /Hap-22 | ― | ― | ― | ― | ― | ― | ― | ― | ― | C | T | G | T | T | T | T | T | |
| *C. e. songaricus* /Hap-23 | ― | ― | ― | ― | A | A | A | T | T | T | T | A | C | T | C | T | C | |
| *C.e. kansuensis* /Hap-24 | ― | ― | ― | ― | A | A | A | T | T | T | T | G | C | T | C | T | T | |
| *C. e. wallichi* /Hap-25 | ― | ― | ― | ― | A | A | A | T | T | T | T | G | C | T | C | T | T | |
| *C.e.manitobensis* /Hap-26 | ― | ― | ― | ― | A | A | A | T | T | T | T | A | T | T | C | T | C | |
| *C.e.nelsoni* /Hap-27 | ― | ― | ― | ― | A | A | A | T | T | T | T | A | T | T | C | T | C | |
| *C.e.roosevelti* /Hap-28 | ― | ― | ― | ― | A | A | A | T | T | T | T | A | T | T | C | T | C | |
| *C.e.xanthopygus* Hap-29 | ― | ― | ― | ― | A | A | A | T | T | T | C | G | T | T | C | T | T | |
| *C. e. alashanicus* /Hap-30 | ― | ― | ― | ― | A | A | A | T | T | T | T | G | C | T | C | T | T | |
| *C.e. sibiricus/*Hap-31 | ― | ― | ― | ― | A | A | A | T | C | T | T | A | T | T | C | T | T | |
| *C. e. canadensis* /Hap-32 | ― | ― | ― | ― | A | A | A | T | T | T | T | G | T | T | C | T | T | |

Continued…

| **Subspecies/Haplotypes** |  | **Variable position** | | | | | | | | | | | | | | |  |
| --- | --- | --- | --- | --- | --- | --- | --- | --- | --- | --- | --- | --- | --- | --- | --- | --- | --- |
| **Nt position** | **15809** | **15810** | **15812** | **15813** | **15814** | **15815** | **15816** | **15817** | **15818** | **15819** | **15820** | **15830** | **15834** | **15835** | **15840** | **15844** | **15846** |
| NC_007704.2 | C | T | C | T | A | T | T | T | A | T | A | A | G | A | G | C | T |
| *C.e. hanglu*/Hap-01 | C | T | T | T | G | T | T | T | A | C | A | A | G | A | G | C | T |
| *C.e. hanglu* /Hap-02 | C | T | T | T | G | T | T | T | A | C | A | A | G | A | G | C | T |
| *C.e. hanglu* /Hap-03 | C | T | T | T | G | T | T | T | A | C | A | A | G | A | G | C | T |
| *C.e. hanglu* /Hap-04 | C | T | T | T | G | T | T | T | A | C | G | A | G | A | G | C | T |
| *C.e. hanglu* /Hap-05 | C | T | T | T | G | T | T | T | A | C | G | A | G | A | G | C | T |
| *C.e. hanglu* /Hap-06 | C | T | T | T | G | T | T | T | A | C | A | A | G | A | G | C | T |
| *C.e. hanglu* /Hap-07 | C | T | T | T | G | T | T | T | A | C | A | A | G | A | G | C | T |
| *C.e. hanglu* /Hap-08 | C | T | T | T | G | T | T | T | A | C | A | A | G | A | G | C | T |
| *C.e. hanglu* /Hap-09 | C | T | T | T | G | T | T | T | A | C | A | A | G | A | G | C | T |
| *C.e. hanglu* /Hap-10 | C | T | T | T | G | T | T | T | A | C | G | A | G | A | G | C | T |
| *C.e. hanglu* /Hap-11 | C | T | T | T | G | T | T | T | A | C | G | A | G | A | G | C | T |
| *C.e. hanglu* /Hap-12 | C | T | T | T | G | T | T | T | A | C | G | A | G | A | G | C | T |
| *C.e. hanglu* /Hap-13 | C | T | T | T | G | T | T | T | A | C | G | A | G | A | G | C | T |
| *C. e. yarkandensis* /Hap-14 | C | C | C | T | G | T | T | T | A | C | A | A | G | A | G | C | T |
| *C. e. bactrianus* /Hap-15 | C | C | C | T | G | T | T | T | A | C | A | A | G | A | G | C | T |
| *C. e. hippelaphus* /Hap-16 | C | T | C | T | A | T | T | T | A | T | A | A | G | A | G | C | T |
| *C. e. hippelaphus* /Hap-17 | C | T | C | T | A | T | T | T | A | T | A | A | G | A | G | C | T |
| *C. e. hispanicus* /Hap-18 | C | T | C | T | A | T | T | T | A | T | A | A | G | A | G | C | T |
| *C. elaphus* /Hap-19 | C | T | C | T | A | T | T | T | A | T | A | A | G | A | G | C | T |
| *C.e. atlanticus* /Hap-20 | C | T | C | T | A | T | T | T | A | T | A | G | G | A | G | C | T |
| *C. e. barbarus* /Hap-21 | C | T | C | T | A | C | T | T | A | T | A | A | G | A | G | T | C |
| *C.e. corsicanus* /Hap-22 | C | T | C | T | A | T | T | T | A | T | A | A | G | A | G | C | T |
| *C. e. songaricus* /Hap-23 | T | C | C | C | A | T | T | C | A | C | A | A | A | G | G | C | T |
| *C.e. kansuensis* /Hap-24 | T | C | C | T | A | T | T | T | A | C | A | A | G | A | A | T | T |
| *C. e. wallichi* /Hap-25 | T | T | C | T | A | T | T | T | A | C | A | A | G | A | A | T | T |
| *C.e.manitobensis* /Hap-26 | T | C | C | T | A | T | C | T | G | C | A | A | A | A | G | C | T |
| *C.e.nelsoni* /Hap-27 | T | C | C | T | A | T | C | T | G | C | A | A | A | A | G | C | T |
| *C.e.roosevelti* /Hap-28 | T | C | C | T | A | T | C | T | G | C | A | A | A | A | G | C | T |
| *C.e.xanthopygus* Hap-29 | C | T | C | T | A | T | C | T | A | C | A | A | A | A | G | C | T |
| *C. e. alashanicus* /Hap-30 | T | C | C | T | A | T | T | T | A | C | A | A | G | A | G | T | T |
| *C.e. sibiricus/*Hap-31 | T | C | C | T | A | T | T | C | A | C | A | A | A | A | G | C | T |
| *C. e. canadensis* /Hap-32 | T | C | C | T | A | T | C | T | A | C | A | A | A | A | G | C | T |

Continued…

| **Subspecies/Haplotype** | **Variable position** | | | | | | | | | | | | | | | |  |
| --- | --- | --- | --- | --- | --- | --- | --- | --- | --- | --- | --- | --- | --- | --- | --- | --- | --- |
| **Nt position** | **15856** | **15857** | **15862** | **15873** | **15875** | **15877** | **15878** | **15893** | **15905** | **15914** | **15920** | **15921** | **15922** | **15923** | **15939** | **15956** | **15960** |
| NC_007704.2 | C | G | A | G | C | C | T | G | C | G | G | A | T | C | A | C | G |
| *C.e. hanglu*/Hap-01 | C | A | A | A | T | C | T | G | C | G | A | A | C | T | A | C | G |
| *C.e. hanglu* /Hap-02 | C | A | T | A | T | C | T | A | C | G | A | A | C | T | A | C | G |
| *C.e. hanglu* /Hap-03 | C | A | T | A | T | C | T | G | C | G | A | A | C | T | A | C | G |
| *C.e. hanglu* /Hap-04 | C | A | A | A | T | C | T | G | C | G | A | A | C | T | A | C | G |
| *C.e. hanglu* /Hap-05 | C | A | A | ― | T | C | T | G | C | G | A | A | C | T | C | C | A |
| *C.e. hanglu* /Hap-06 | C | A | A | A | T | C | T | G | C | G | A | A | C | T | C | C | A |
| *C.e. hanglu* /Hap-07 | C | A | A | A | T | C | T | G | C | G | A | A | C | T | C | C | A |
| *C.e. hanglu* /Hap-08 | C | A | A | ― | T | C | T | G | C | G | A | A | C | T | A | G | G |
| *C.e. hanglu* /Hap-09 | C | A | A | A | T | C | T | G | C | G | A | A | C | T | A | C | G |
| *C.e. hanglu* /Hap-10 | C | A | A | A | T | C | T | G | C | G | A | A | C | T | C | C | G |
| *C.e. hanglu* /Hap-11 | C | A | A | A | T | C | T | G | C | G | A | A | C | T | A | C | G |
| *C.e. hanglu* /Hap-12 | C | A | A | A | T | C | T | G | C | G | A | A | C | T | C | C | G |
| *C.e. hanglu* /Hap-13 | C | A | A | A | T | C | T | G | C | G | A | A | C | T | C | C | A |
| *C. e. yarkandensis* /Hap-14 | C | G | A | A | T | C | T | G | C | G | A | A | C | T | A | C | G |
| *C. e. bactrianus* /Hap-15 | T | G | A | G | T | T | T | G | C | G | A | A | C | T | A | C | G |
| *C. e. hippelaphus* /Hap-16 | C | G | A | A | T | T | T | A | C | G | G | A | T | C | A | C | G |
| *C. e. hippelaphus* /Hap-17 | T | G | A | A | T | C | T | G | C | G | G | A | T | C | A | C | G |
| *C. e. hispanicus* /Hap-18 | T | G | A | G | C | C | T | G | T | G | A | A | T | T | A | C | G |
| *C. elaphus* /Hap-19 | C | G | A | G | C | T | T | G | C | G | G | A | T | C | A | C | G |
| *C.e. atlanticus* /Hap-20 | C | G | A | G | C | T | T | G | C | G | G | A | T | T | A | C | G |
| *C. e. barbarus* /Hap-21 | C | G | A | G | C | T | T | G | C | G | G | G | T | C | A | C | G |
| *C.e. corsicanus* /Hap-22 | T | A | A | G | C | T | T | G | C | G | G | G | T | C | A | C | G |
| *C. e. songaricus* /Hap-23 | C | A | A | G | C | T | C | G | C | G | A | A | T | T | A | C | G |
| *C.e. kansuensis* /Hap-24 | C | A | A | G | C | T | T | G | C | G | A | A | T | T | A | C | G |
| *C. e. wallichi* /Hap-25 | T | G | A | G | C | T | T | G | C | G | A | A | T | T | A | C | G |
| *C.e.manitobensis* /Hap-26 | C | G | A | G | C | T | T | G | C | G | A | A | T | T | A | C | G |
| *C.e.nelsoni* /Hap-27 | T | A | A | G | C | T | T | G | C | G | A | A | T | T | A | C | G |
| *C.e.roosevelti* /Hap-28 | T | A | G | G | C | T | T | G | C | A | A | A | T | T | A | C | G |
| *C.e.xanthopygus* Hap-29 | T | A | A | G | C | T | T | G | C | G | A | A | T | T | A | C | G |
| *C. e. alashanicus* /Hap-30 | C | G | A | G | C | T | T | G | C | G | A | A | T | T | A | C | G |
| *C.e. sibiricus/*Hap-31 | T | G | A | G | C | C | T | G | C | G | A | A | T | T | A | C | G |
| *C. e. canadensis* /Hap-32 | T | A | A | G | C | T | T | G | C | G | A | A | T | T | A | C | G |

Continued…

| **Subspecies/Haplotypes** | **Variable position** | | | | | | | | | | | |  |
| --- | --- | --- | --- | --- | --- | --- | --- | --- | --- | --- | --- | --- | --- |
| **Nt position** | **15963** | **15969** | **15974** | **15975** | **15985** | **15986** | **15987** | **15989** | **15990** | **15922** | **16004** | **16006** | **16007** |
| NC_007704.2 | C | T | C | T | T | G | A | C | T | T | T | T | A |
| *C.e. hanglu*/Hap-01 | C | T | C | T | T | A | A | T | T | T | C | T | A |
| *C.e. hanglu* /Hap-02 | C | T | C | T | T | A | A | ― | T | G | C | T | A |
| *C.e. hanglu* /Hap-03 | C | T | C | T | T | A | A | T | T | G | C | T | A |
| *C.e. hanglu* /Hap-04 | C | T | C | C | A | A | A | T | T | G | C | T | A |
| *C.e. hanglu* /Hap-05 | C | T | C | C | A | A | A | ― | T | G | C | T | A |
| *C.e. hanglu* /Hap-06 | C | T | C | T | A | A | A | ― | T | G | C | T | A |
| *C.e. hanglu* /Hap-07 | C | T | C | C | A | A | A | T | T | G | C | T | A |
| *C.e. hanglu* /Hap-08 | C | C | G | T | A | A | A | T | T | G | C | T | A |
| *C.e. hanglu* /Hap-09 | C | C | G | T | A | A | A | T | T | G | C | T | A |
| *C.e. hanglu* /Hap-10 | C | T | C | T | T | A | A | T | T | T | C | T | A |
| *C.e. hanglu* /Hap-11 | C | T | C | T | T | A | A | T | T | T | C | T | A |
| *C.e. hanglu* /Hap-12 | C | T | G | C | A | A | A | T | T | G | C | T | A |
| *C.e. hanglu* /Hap-13 | C | T | C | C | A | A | A | T | T | G | C | T | A |
| *C. e. yarkandensis* /Hap-14 | C | T | C | T | T | A | A | T | T | T | T | T | A |
| *C. e. bactrianus* /Hap-15 | T | T | C | T | T | A | G | T | C | T | T | T | A |
| *C. e. hippelaphus* /Hap-16 | C | T | C | T | T | A | A | T | T | T | T | T | A |
| *C. e. hippelaphus* /Hap-17 | C | T | C | T | T | G | A | C | T | T | T | T | A |
| *C. e. hispanicus* /Hap-18 | C | T | C | T | T | G | A | C | T | T | T | T | A |
| *C. elaphus* /Hap-19 | C | T | C | T | T | G | A | T | C | T | T | T | A |
| *C.e. atlanticus* /Hap-20 | C | T | C | T | T | G | A | C | T | T | T | T | A |
| *C. e. barbarus* /Hap-21 | C | T | C | T | T | A | A | T | C | T | T | T | A |
| *C.e. corsicanus* /Hap-22 | C | T | C | T | T | A | G | T | C | T | T | T | A |
| *C. e. songaricus* /Hap-23 | C | T | T | T | T | G | A | C | C | T | T | T | T |
| *C.e. kansuensis* /Hap-24 | C | T | C | T | T | G | A | C | C | T | T | T | A |
| *C. e. wallichi* /Hap-25 | C | T | C | T | T | G | A | C | C | T | T | T | A |
| *C.e.manitobensis* /Hap-26 | C | T | C | T | T | G | A | C | C | T | T | T | A |
| *C.e.nelsoni* /Hap-27 | C | T | C | T | T | G | A | C | C | T | T | T | A |
| *C.e.roosevelti* /Hap-28 | C | T | C | T | T | G | A | C | C | T | T | T | A |
| *C.e.xanthopygus* Hap-29 | C | T | C | T | T | G | A | C | C | T | C | C | A |
| *C. e. alashanicus* /Hap-30 | C | T | C | T | T | G | A | C | C | T | T | T | A |
| *C.e. sibiricus/*Hap-31 | C | T | C | T | T | A | A | C | C | T | T | T | A |
| *C. e. canadensis* /Hap-32 | C | T | C | T | T | G | A | C | C | T | T | T | A |
