# Supplementary material for: Pragmatic Perspective on Conservation Genetics and Demographic History of the Last Surviving Population of Kashmir Red Deer (Cervus elaphus hanglu) in India
Source: PLoS One. 2015 Feb 11;10(2):e0117069. doi: 10.1371/journal.pone.0117069 (PMC4324630; doi:10.1371/journal.pone.0117069)
Supplement: S4 Table — (DOCX) [file pone.0117069.s004.docx]

**Supporting table S4-** Summary of paramters used in the model the senarios; EV= environmental variation, expressed as a standard deviation.

| **Parameter** | **Values** |
| --- | --- |
| Breeding system | Polygny |
| Age of first reproduction (male/female) in years | 3/4 |
| Maximum age (in years) | 20 |
| Age of seniscance (in years) male/female | 17/18 |
| % adult males in breeding pool | 30 (10) |
| Annual % adult females reproducing (EV) | 50 (5) |
| Average and maximum litter size | 1 |
| Overall offspring sex ratio | 50:50 |
| % mortaility from are 0-1 (EV) (male/female) | 50 (10)/ 50 (10) |
| % mortaility from are 1-2 (EV) (male/female) | 10(3)/10(3) |
| % mortaility from are 2-3 (EV) (male/female) | 10(3)/10(3) |
| % mortaility above 3 (EV) (male/female) | 10(3)/10(3) |
| Inbreeding depression | 0.38 (senario-I) /  6.14 (senario-II) |
| % of inbreeding effect due to recessive lethal alleles | 50 |
| Carrying capacity (EV) | 300 (30) |
| Initial population size | 218 |
