# Supplementary material for: Pragmatic Perspective on Conservation Genetics and Demographic History of the Last Surviving Population of Kashmir Red Deer (Cervus elaphus hanglu) in India
Source: PLoS One. 2015 Feb 11;10(2):e0117069. doi: 10.1371/journal.pone.0117069 (PMC4324630; doi:10.1371/journal.pone.0117069)
Supplement: S1 Fig — (DOCX) [file pone.0117069.s005.docx]

**Supporting figure S1- Pairwise mismatch distribution using mitochondrial D loop sequences of *hangul* population** (the observed multimodal distribution pattern is an indicator of the population to be under demographic equilibrium).
