# Supplementary material for: Pragmatic Perspective on Conservation Genetics and Demographic History of the Last Surviving Population of Kashmir Red Deer (Cervus elaphus hanglu) in India
Source: PLoS One. 2015 Feb 11;10(2):e0117069. doi: 10.1371/journal.pone.0117069 (PMC4324630; doi:10.1371/journal.pone.0117069)
Supplement: S2 Fig — (DOC) [file pone.0117069.s006.doc]

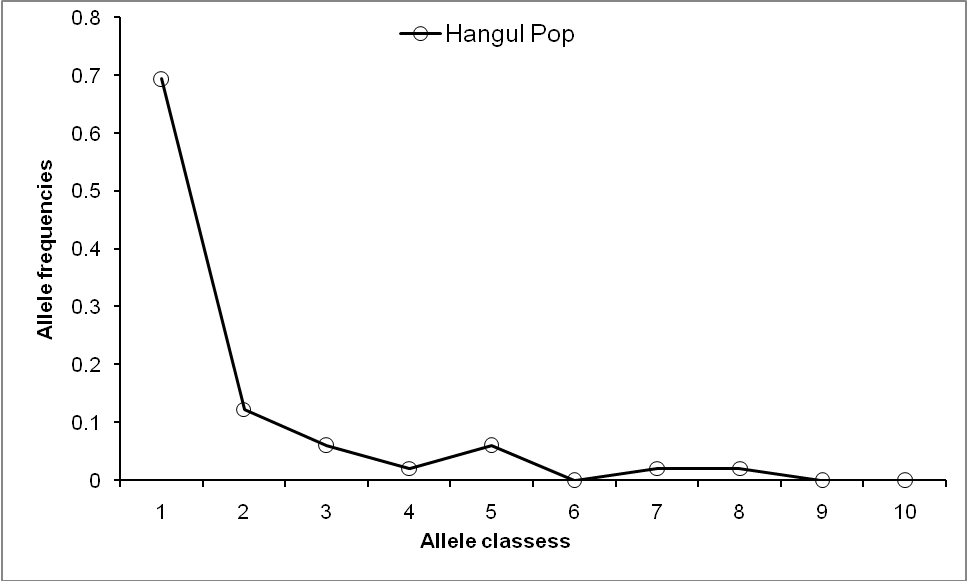


**Supporting figure S2- Qualitative “L‐shaped mode‐shift” allele distribution test for bottleneck analysis of *hangul* populations**
